# Development and in vitro evaluation of biomimetic injectable hydrogels from decellularized human nerves for central nervous system regeneration

**DOI:** 10.1016/j.mtbio.2025.101483

**Published:** 2025-01-11

**Authors:** Gopal Agarwal, Kennedy Moes, Christine E. Schmidt

**Affiliations:** J. Crayton Pruitt Family Department of Biomedical Engineering, Herbert Wertheim College of Engineering, University of Florida, Gainesville, FL, 32611, USA

**Keywords:** Injectable hydrogel, Natural biomaterials, Human peripheral nerves, Decellularization, Delipidation, Neural tissue regeneration, CNS tissue biomimetics

## Abstract

Injuries to the central nervous system (CNS) often lead to persistent inflammation and limited regeneration. This study developed a clinically relevant injectable hydrogel derived from decellularized human peripheral nerves, with mechanical properties biomimicking native CNS tissue. Using a modified Hudson method, human sciatic nerves were decellularized, effectively removing immunogenic cellular debris while retaining the extracellular matrix. Two delipidation solvents, dichloromethane: ethanol (2:1 v/v) and n-hexane: isopropanol (3:1 v/v), were evaluated, with the former achieving optimal lipid removal and better digestion. The resulting solution was crosslinked with genipin, forming an injectable hydrogel (iHPN) that gelled within 12 min at 37 °C and exhibited mechanical stiffness of approximately 400 Pa. Human astrocytes, human microglial cell clone 3 (HMC3), and mouse RAW 264.7 macrophages were cultured individually within iHPN, with lipopolysaccharide (LPS) added to mimic CNS inflammation following injury. Compared to LPS-activated cells on tissue culture plates (TCP), astrocytes within iHPN maintained a quiescent state, as evidenced by reduced GFAP expression and IL-1β secretion. RAW 264.7 and HMC3 cells in iHPN displayed an anti-inflammatory phenotype, as shown by increased CD206 and decreased CD86/CD68 expression, along with higher IL-4 and lower TNF-α/IL-1β secretion. Human SH-SY5Y neuroblastoma cells exhibited higher viability and improved neuronal differentiation in iHPN compared to TCP. Human brain neurons had higher neuronal differentiation within iHPN compared to TCP or collagen hydrogels. Overall, iHPN is a novel injectable hydrogel that has potential for minimally invasive CNS applications, such as a carrier for cell or drug delivery and/or a biomaterial to support axonal growth.

## Graphical abstract

**Figure:**
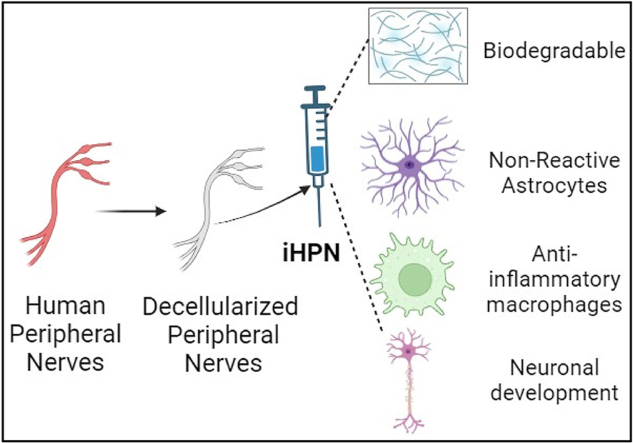

## 1 Introduction

Central nervous system (CNS) injuries, including traumatic brain injury, stroke, and spinal cord injury (SCI), are leading causes of long-term disability. Unfortunately, the limited regeneration capability of CNS neurons leads to permanent functional loss [1], posing a major challenge in the treatment of CNS injuries and disorders. Macrophages/microglial cells and astrocytes play a crucial role in neuroinflammatory processes, regulating the immune response in the CNS [2]. Macrophages and microglial cells can acquire distinct phenotypes in response to different microenvironmental cues. They can adopt either a ‘classically activated’ (M1) phenotype, which is pro-inflammatory and cytotoxic, releasing destructive pro-inflammatory mediators, or an ‘alternatively activated’ (M2) phenotype, which is anti-inflammatory and supports tissue repair by clearing cellular debris through phagocytosis and releasing numerous protective and trophic factors [3,4]. Therefore, it remains crucial to develop therapeutic strategies that can modulate astrocyte and macrophage/microglia reactivity at the site of injury [5].

Over the past decade, the rapidly advancing field of biomaterials has introduced promising new treatments for CNS regeneration in preclinical studies [[6], [7], [8], [9], [10]]. Following injury to soft tissues like the CNS, an appropriate scaffold is needed to replicate the natural structural, chemical, and mechanical properties of the extracellular matrix (ECM). Scaffolds should also minimize immune responses, help prevent glial scarring-induced CNS damage, and promote neurite regeneration [6]. Decellularization of tissue creates non-immunogenic, native ECM scaffolds that can enhance tissue regeneration [7,8]. Decellularized extracellular matrix (dECM) materials have been used for over 20 years as regenerative biomaterial scaffolds due to their unique biological activity and excellent biocompatibility [9,10]. Decellularized scaffolds are immunologically tolerated and maintain native structures, tissue cell adhesion proteins, growth factors, and glycosaminoglycans, which promote proper tissue remodeling in the host [9,10]. Hydrogels derived from decellularized tissues have gained significant attention for their injectability, tunable mechanical properties, and ease of modification with bioactive molecules [11]. Previously, our lab developed an efficient decellularization method, the Hudson method [12], for peripheral nerves, which was licensed to Axogen Inc. as part of their process for creating the AVANCE™ nerve graft product. To date, the product has been successfully implanted in over 100,000 patients with peripheral nerve injuries, leading to improved functional motor recovery [[13], [14], [15]]. Peripheral nerve grafts have been long recognized for their ability to support axonal regeneration and promote behavioral improvement in CNS injuries, such as SCI. These grafts offer a native, biomimetic environment rich in growth-promoting factors and ECM components that facilitate axonal extension, remyelination, and synaptic reformation [16,17]. Additionally, peripheral nerve grafts act as bridges, connecting the damaged nerve ends and guiding regenerating axons, while also modulating the immune response to create a more favorable environment for neural repair [18]. However, a limitation of implanting an intact scaffold, such as whole nerve tissue, is the need for a surgical procedure to excise enough tissue from the affected area to allow for the insertion of the construct [19]. In addition, the pre-formed structure of intact scaffolds can complicate integration within irregularly shaped lesion cavities [19]. To address these limitations in CNS applications, this study developed an injectable hydrogel using decellularized human peripheral nerves (iHPN). This novel injectable formulation enables its use in applications where minimally invasive injection is preferred or required [20,21]. Injectable materials also provide the advantage of a straightforward approach to combining cells, biomolecules, and drugs as delivery vehicles for different treatment options [22,23].

Various chemical and enzyme-based decellularization methods have been explored for human peripheral nerves in the past. For instance, Suss et al. [24] utilized a combination of sonication and Triton X-100 detergents for decellularization. Bae et al. [25] incorporated (3-((3-cholamidopropyl) dimethylammonio)-1-propanesulfonate) (CHAPS) detergent along with DNase and RNase A enzyme treatments for decellularization of human peripheral nerve allograft. Nicolau et al. [26] used a mixture of sulfobetaine 10 and 16, Triton X-100, 1 M NaCl, and Pulmozyme DNase for decellularization of human peripheral nerves. While these protocols have shown varying degrees of success, they have two main limitations. First, they fail to remove residual lipids and proteoglycans, which can negatively impact tissue regeneration by inducing inflammatory responses and inhibiting axonal growth [27]. Additionally, these methods focus on preparing decellularized grafts rather than injectable hydrogels, which offer minimally invasive application and greater adaptability for neural tissue regeneration. Therefore, in this study, we have modified our lab's peripheral nerve decellularization protocol, the Hudson method [12], to effectively remove cellular constituents while retaining ECM in human nerves. An optimal delipidation solvent was selected from two different organic solvents. The resultant dECM was used to fabricate injectable hydrogels, ideal for minimally invasive applications, such as those commonly required in the CNS.

**Fig. 1:**
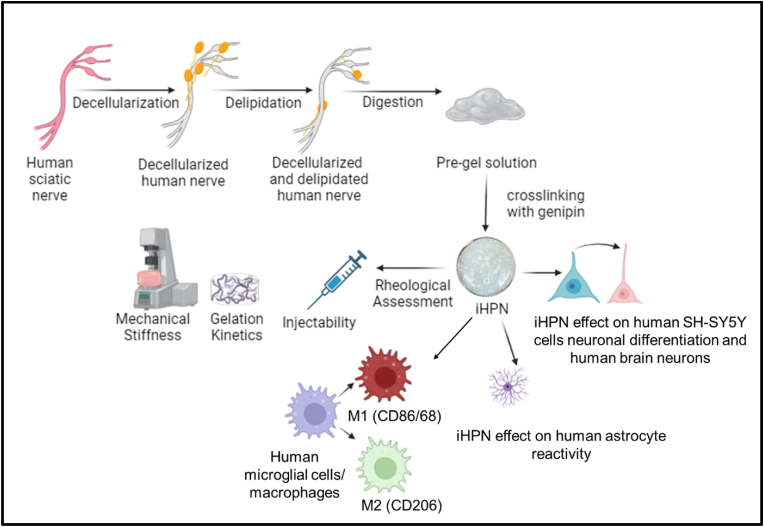
**Overall experimental flow to create and evaluate injectable human peripheral nerve (iHPN)**: Peripheral nerves obtained from a human tissue bank were decellularized by modifying our previously published Hudson method [12,31]. As human nerves contain sufficient lipids to prevent efficient gelation, the decellularized nerves were delipidated using dichloromethane and ethanol solvent and digested using pepsin to form a pre-gel injectable solution. The hydrogel solution was then crosslinked with genipin to strengthen the final gels. The injectability, gelation kinetics (to determine the time taken for hydrogel solution to gel), and frequency sweep (to measure the mechanical stiffness of hydrogels) were assessed using a rheometer. The effects of iHPN on astrocyte reactivity, microglial/macrophage polarization, and neuronal differentiation of SH-SY5Y neuroblastoma cells/human brain neurons were evaluated *in vitro (Image credit:*BioRender.com*)*.

Our lab has previously shown that injectable hydrogel derived from decellularized rat sciatic nerves can provide pro-regenerative cues, promote an anti-inflammatory phenotype in macrophages/microglial cells, and serve as a cell delivery vehicle in a rat model of SCI [12,[28], [29], [30], [31]]. However, the use of a decellularized rat matrix limits future clinical translation. To address this, we developed an injectable hydrogel using decellularized human peripheral nerves. Developing injectable hydrogels using human tissue, unlike rat tissue, presents challenges due to the high concentrations of lipids. We have previously shown that the lipid remnants in decellularized porcine peripheral nerves, which have a composition similar to human nerves, hinder the gelation of hydrogels, affecting their mechanical stiffness [32]. Although porcine nerves offer potential for clinical translation, the fabrication of injectable hydrogels using decellularized human peripheral nerves has a higher likelihood of successful clinical application for neural tissue regeneration. Therefore, in this study, we modified the Hudson method [12] by replacing Triton X-200 with Triton X-100, because Triton X-200 was discontinued; furthermore, we incorporated incubations with DNAse (degradation of nuclear DNA) and chondroitinase ABC (digestion of chondroitin sulfate proteoglycans) for decellularizing human sciatic nerves [31]. We also added a delipidation step using a dichloromethane and ethanol solvent, which effectively removed lipid remnants from decellularized nerves. The impact of lipid remnants on the gelation kinetics of injectable hydrogels was studied. The resulting decellularized and delipidated human sciatic nerves were then used to fabricate injectable hydrogel formulations. Additionally, we examined the effect of genipin-mediated chemical crosslinking on iHPN formulations to adjust the mechanical strength of the hydrogels. We designed hydrogels with a mechanical stiffness around 400 Pa, mimicking the mechanical strength of CNS tissue [33,34] and being sufficient to promote neural tissue regeneration [35]. The efficacy of the developed injectable hydrogel formulations in providing pro-regenerative cues for neuronal differentiation was evaluated by analyzing the differentiation of SH-SY5Y cells (a neuroblastoma cell line) and human brain neurons into neuronal phenotypes. Finally, we further explored the effect of iHPN on the reactivity of human astrocytes and macrophages/microglia (Fig. 1). Lipopolysaccharide (LPS), a component of gram-negative bacteria cell walls, stimulates astrocytes by triggering toll-like receptor (TLR) 4 signaling, which activates nuclear factor kappa-B (NF-ƘB) [36]. LPS-stimulated astrocytes show increased expression of glial fibrillary acidic protein (GFAP) and secrete inflammatory cytokines, including interleukin (IL)-1β [37]. LPS also stimulates macrophages/microglial cells via TLRs, leading to the activation of NF-ƘB, mitogen-activated protein kinase (MAPK), and other inflammatory signaling pathways [38]. This causes excessive release of inflammatory mediators and cytokines, such as nitric oxide (NO), tumor necrosis factors (TNF-α), IL-1β, IL-6, and IL-10, triggering a series of abnormal inflammatory responses [39,40]. Macrophages/microglial cells can also polarize either toward the M1 phenotype, which is pro-inflammatory and characterized by high CD86 or CD68 [41] expression, or toward the M2 phenotype, which is anti-inflammatory and marked by high mannose receptor (CD206) expression depending on microenvironmental cues [42].

In this study, human astrocytes were cultured within iHPN and LPS was used to induce inflammatory microenvironment. The effect of iHPN on human astrocytes' reactivity was assessed by immunostaining for GFAP marker, measuring IL-1β cytokines levels. To study iHPN's effect on macrophages polarization, RAW 264.7 cells, a macrophage-like murine cell line was used. RAW 264.7 cells demonstrate key functions similar to human macrophages, including phagocytosis, pinocytosis, and the ability to respond to inflammatory stimuli [43]. Therefore, we used RAW 264.7 cells to assess the effect of iHPN on macrophage polarization in the presence of MC3the LPS stimulus. Immunostaining was performed for two distinct markers: CD206 and CD86. Additionally, cytokine secretions like TNF-α, IL-4, and IL-10 and NO levels from RAW 264.7 cells cultured within iHPN were estimated using ELISA. Similarly, iHPN's effect on microglial cells was examined by culturing human microglial cells clone 3 (HMC3) within iHPN and activating them using LPS. The cytokines (TNF-α, IL-1β and IL-4) levels were estimated using ELISA and inflammatory phenotype was assessed using the CD68 marker.

Overall, this study presents the development of an injectable hydrogel formulation derived from decellularized human peripheral nerves, with the potential to be used as part of treatment therapies for CNS disorders.

## 2 Materials and methods

### 2.1 Human sciatic nerve decellularization

Deidentified human sciatic nerves were procured from the National Disease Research Interchange (NDRI) from six different donors. The nerves were kept at −80 °C in a biohazard bag until further use. Decellularization of the sciatic nerves was adapted for human tissue by modifying our lab's previously published Hudson protocol [12,31]. Briefly, nerves were subjected to 7 h of washing in water, followed by 18 h of washing in 125 mM sulfobetaine (SB-10) buffer. Thereafter, the nerves were washed with 100 mM Na/10 mM phosphorus buffer for 15 min, and then with 3 % sodium deoxycholate (SD)/0.14 % Triton X-100 solution for 24 h, followed by washing the nerves with 50 mM Na/10 mM phosphorus buffer three times, for 15 min each. The nerves were again treated with SB-10 buffer (for 7 h) and SD/Triton X-100 solution (for 15 h). To remove any residual DNA content, the nerves were incubated in 75 U/mL of DNAase solution for 3 h. The nerves were then washed once with 0.2 U/ml of chondroitinase ABC enzyme solution for 24 h. Two different organic solvent-based delipidation protocols were assessed (i) hexane: isopropanol (3:1 v/v); and (ii) dichloromethane: ethanol (2:1 v/v) to assess their efficacy for reducing residual lipids. The nerves were treated with delipidation solvents for 24 h. Additionally, some nerves were treated twice with dichloromethane: ethanol (2:1) for an additional 24 h. Finally, the nerves were washed with water for 72 h, changing water every 24 h. The nerves that were decellularized only and not subjected to any delipidation steps are denoted as “decell only”, whereas those nerves subjected to dichloromethane: ethanol solvent treatment are represented as “decell + delipid”.

### 2.2 Immunohistochemistry analysis

After decellularization and delipidation, nerves were fixed using 4 % paraformaldehyde (PFA) and stored in 30 % sucrose for 1 week. The nerves were then trimmed, soaked in optimal cutting temperature (OCT) compound at 4 °C overnight, and flash frozen at −80 °C for at least 5 min. OCT blocks were then stored at −80 °C until sectioning. Frozen sections, 20 μm thick, were obtained using a Leica CM1950 cryostat (Leica Biosystems, Germany). Sections were stained with antibodies against collagen I (Millipore Sigma; C2456, 1:500), collagen IV (Abcam; ab6586, 1:500), laminin (Millipore Sigma; L9393, 1:500), and 4′,6-diamidino-2-phenylindole (DAPI) for nuclei (Thermo Fisher Scientific; D1306, 1:1000). Secondary antibodies conjugated with Alexa Fluor 488/647 (2 μg/mL) were incubated on sections for 1 h at room temperature (RT). Negative controls (without primary antibody) were prepared to ensure minimal autofluorescence and nonspecific staining, while native nerves were used as positive controls. For each donor tissue, two consecutive sections were stained for each marker. The sections were imaged using a Zeiss 880 confocal microscope.

### 2.3 dsDNA quantification assessment

Nerves were lyophilized for 72 h, and the tissue was weighed. The nerves were then digested using 0.5 mg/mL papain from papaya latex (Sigma Aldrich; P3125-25 mg) overnight at 60 °C. The isolated DNA was then quantified using Quant-iT PicoGreen™ dsDNA reagent kit (Invitrogen; P11496) according to the manufacturer's protocol. The DNA amount was calculated by extrapolating from a standard curve using lambda DNA.

### 2.4 Endotoxin estimation

The endotoxin level in our decellularized and delipidated iHPN solution was determined using the Pierce™ Chromogenic Endotoxin Quant kit (Thermo Fischer, A39553). Briefly, the decellularized and delipidated nerves were digested using 0.5 mg/mL of papain enzyme as explained earlier. The digested nerve solution was used to quantify endotoxin levels using Pierce™ Chromogenic Endotoxin Quant kit as per the manufacturer's recommendations. The endotoxin level was calculated by extrapolating from a standard curve using the *E. coli* endotoxin standard.

### 2.5 Oil Red O staining for lipid estimation in decellularized nerves

Cryosectioned human nerve slices (n = 2 each from three different donors) were used for Oil O Red staining. Briefly, the sections were incubated in 60 % isopropanol for 5 min, followed by incubation in Oil O Red stain for 15 min. The sections were washed with water three times and then counterstained with Hematoxylin for 1 min. The sections were subsequently washed twice with water and mounted with Fluoromount aqueous medium (Sigma Aldrich; F4680) using a coverslip. The sections were imaged under a compound microscope (Leica MC 170 HD) at 4x, 10x, or 20× objective, and images were captured.

### 2.6 Lipid estimation in decellularized nerves

The lipid content in decellularized nerves was estimated using a Lipid Assay Kit (unsaturated fatty acids) from Abcam (catalog number: ab242305). This assay allows for the measurement of lipid content (unsaturated fatty acids only) using sulfo-phospho vanillin reagent through colorimetric absorbance. Lyophilized nerves (native, decell only, and decell + delipid, n = 3 from three different donors) were digested using 0.5 mg/mL papain from papaya latex (Sigma Aldrich; P3125-25 mg) overnight at 60 °C. The white lipid-rich solution obtained after papain digestion was used for lipid quantification. Briefly, 15 μL of solution was placed into a 96-well plate and then incubated at 90 °C for 30 min. Samples were then cooled to 4 °C for 5 min, and 150 μL of 18 M sulfuric acid was added to each well. After a 10-min incubation at 90 °C, the samples were cooled to 4 °C for 5 min. A 100 μL sample was transferred to a clean 96-well plate, and background absorbances were measured at 540 nm. Sulfo-phospho vanillin reagent (100 μL) was added to each well, and mixed thoroughly, incubated at 37 °C for 15 min, and absorbance was measured at 540 nm. The background absorbance was subtracted from each well. The concentrations of lipids in the samples were determined by extrapolating from a standard curve, which was plotted using the lipid standard supplied by the manufacturer.

### 2.7 Decellularized nerve digestion

Decellularized/delipidated sciatic nerves were solubilized using a previously published method [32]. Lyophilized nerves were weighed and minced into small pieces in a scintillation vial (Millipore Sigma). A 1 mg/mL pepsin (Sigma Aldrich; P7012) solution in 0.01 M hydrochloric acid (Sigma Aldrich; 258,148) was added to achieve tissue concentrations of 15 mg of tissue per 1 mL of pepsin solution. Vials were capped and incubated for 72 h at RT while under constant agitation with a magnetic stir bar. Undigested nerve components were separated by centrifuging at 3000×*g* for 15 min at 4 °C. The supernatants of the nerve digests, now homogenous solutions, were transferred into microcentrifuge tubes and placed on ice. To neutralize the solutions, 1 M NaOH was added to the digests until the pH reached 7.4. The solutions were stabilized by adding 10X PBS in an amount equal to 1/9th of the volume of the digests. The neutralized solutions are referred to as “pre-gel” solutions and were used for protein estimation, mammalian cell culture, and rheological analysis, as explained below.

### 2.8 Efficiency of pepsin to digest human nerve

To analyze the efficiency of pepsin in digesting decellularized nerves, the undigested tissue pieces after 72 h of pepsin digestion were freeze-dried, and the dry weight of the undigested tissue was recorded. The percentage of dry weight of undigested tissue was calculated as follows:

[1] %dryweightundigestedtissue=(originalweightoftissuetaken−weightofundigestedtissue)/(originalweightoftissuetaken)∗100

### 2.9 Collagen estimation

The undigested tissue solution after pepsin digestion was centrifuged at 1500×*g* for 2 min at 4 °C. The collected supernatant was used for total collagen estimation using a Collagen Assay Kit (Sigma Aldrich; MAK322). Briefly, the collagen-containing solution was digested into peptides. Subsequently, the N-terminal glycine-containing peptide was reacted with the dye reagent provided by the manufacturer to form a fluorescent complex. The fluorescence intensity of the resultant product was measured at 375 nm excitation and 465 nm emission, which was directly proportional to collagen concentration in the sample. The collagen concentration was extrapolated using the standard collagen solution provided by the manufacturer.

### 2.10 Crosslinking of decellularized nerve pre-gel solution

Pre-gel solutions were crosslinked individually using different concentrations of genipin (Sigma Aldrich; G4796). Various concentrations of genipin (dissolved in DMSO, as stock 89 mM) were added to 15 mg/mL neutralized pre-gel solutions to a final concentration of 2.5 and 5 mM genipin and incubated in a 37 °C incubator chamber to undergo gelation.

### 2.11 Rheological analysis

A time sweep was performed to assess the gelation time of the hydrogel at 37 °C [44]. The mechanical stiffness of the hydrogel was evaluated using a frequency sweep, which measured the storage modulus (G′) and loss modulus (G″) [45]. Additionally, the decrease in viscosity with increasing shear strain demonstrated the hydrogel's shear-thinning behavior, which is a key property indicative of its injectability [44]. In this study, time sweep, frequency sweep, and shear thinning experiments were carried out using an Anton Paar MCR 302 rheometer with a 1 mm gap and an 8 mm sandblasted parallel plate. For time sweep studies, 125 μL of neutralized pre-gel solution with/without genipin was placed on a 37 °C pre-warmed Peltier plate with a 1 mm gap at 1 % shear strain and 1 Hz frequency. G′ and G″ were recorded at 10-s intervals up to 1000 s. For frequency sweep studies, the hydrogels were cast in 8 mm silicone molds by pouring 125 μL of pre-gel neutralized solution with/without genipin crosslinker. The frequency varied from 0.01 to 100 Hz at a constant 1 % strain at 25 °C while G′ and G″ were recorded. Shear thinning experiments were conducted by placing 125 μL of neutralized pre-gel solution with/without crosslinker; shear rate varied from 0 to 150 Pa at 25 °C, while viscosity was recorded.

### 2.12 Injectability assessment

To evaluate the injectability of the hydrogel solution, a ∼15 mg/mL neutralized pre-gel solution was prepared and mixed with 5 mM genipin. The mixture was drawn into syringes fitted with 25 G, 27 G, and 30 G needles at RT. The solution was first withdrawn from a microcentrifuge tube into the syringe and then passed through the needle into a petri dish. The process of drawing the solution into the syringe and subsequently extruding it through the needle was recorded to assess ease of injectability.

### 2.13 In vitro human SH-SY5Y cell biocompatibility and neuronal differentiation

SH-SY5Y cells are a cloned subline of a neuroblastoma cell line derived from a metastatic bone tumor, and they can undergo differentiation into a neuronal phenotype when treated with retinoic acid [46]. Human SH-SY5Y cells (ATCC), P3-P5 were cultured in DMEM, 15 % FBS, 1 % Penicillin-Streptomycin (Pen-Strep) solution at 37 °C, 5 % CO_2_. The media was changed every 2–3 days, and the cells were trypsinized when the cell confluency reached around 70–80 %. For estimating the viability of cells, nearly 50,000 cells were cultured within iHPN hydrogels for 3 days. Thereafter, live/dead stain (LIVE/DEAD™ Viability/Cytotoxicity Kit, for mammalian cells, L3224, Invitrogen) was added and incubated for 30 min at RT. The cells were then imaged using a Zeiss 880 confocal microscope. The live/dead cell ratio was then analyzed using Image J software. Briefly, the images were exported to Image J software, converted to 8 bits, and the same threshold values were applied to all images. The numbers of cells were then counted using the Image J software Analyze toolbar. The ratio of live/dead cells was subsequently calculated. For neuronal differentiation, approximately 50,000 cells were added to iHPN (n = 3 from different donors), and differentiation was induced using DMEM, 3 % FBS, 10 μM retinoic acid, and 1 % Pen-Strep. The neuronal differentiation media was changed every 2 days for 2 weeks of culturing. After that, the cells were fixed and processed for β III tubulin immunostaining to visualize the microtubule network [47], as explained in section 2.19 below. Cells cultured in 24-well tissue plastic (TCP) plates, at the same initial seeding density, served as controls.

### 2.14 In vitro human brain neurons differentiation

Primary human brain neurons (P0; ScienCell; 1520) were cultured in a complete neuron medium (ScienCell; 1521) according to the manufacturer's instructions. iHPN was prepared and neutralized as described previously. Approximately 25,000 neurons were suspended in the hydrogel solution, and 5 mM genipin was added as a crosslinking agent. The mixture was incubated at 37 °C in a 5 % CO₂ atmosphere for 30 min to ensure complete gelation. The formed hydrogels were maintained in a complete neuronal medium, with media changes performed every 2 days for 14 days. At the end of the culture period, the hydrogels were fixed with 4 % PFA and immunostained for β-tubulin III to evaluate neuronal differentiation. Cells seeded at an equivalent density on TCP and within Col hydrogels served as controls.

### 2.15 In vitro human astrocyte cell culturing

Astrocytes are a sub-type of glial cells in the CNS that, in addition to playing essential roles in healthy tissue, become reactive following CNS injury and can hinder regeneration. Therefore, minimizing astrocyte reactivity is often a key goal in therapeutic applications for CNS injury [48,49]. Neonatal human astrocyte cells (Lonza; CC-2565), P 1–3 were cultured in complete astrocyte medium having 10 % FBS and 1 % Pen-Strep as provided by the manufacturer. The cells were maintained at 37 °C and 5 % CO_2_, with medium changes every 2–3 days. When the cell's confluency reached around 70–80 %, cells were trypsinized. Approximately, 1 × 10^5^ cells/group were seeded within the iHPN hydrogels (n = 3 from different donors). The media was changed every 2 days. Following that, the cells were fixed and processed for immunostaining of GFAP as explained below. The upregulation of GFAP, one of type III intermediate filaments and an astrocyte-specific biomarker denotes that astrocytes are activated [50]. In this study, for controls, rat tail collagen type I solution (Corning; 354249) at a concentration of ∼10 mg/mL was neutralized using 1 N NaOH and 10x PBS. The same initial density of cells as in iHPN was added to the neutralized collagen solution and genipin (final concentration of 5 mM) was added to the collagen solution (Col). Cells cultured in 24-well TCP plates, at the same initial seeding density, served as controls. To induce astrocyte activation, a positive control group of cells was seeded in 24-well TCP plates at the same initial seeding density and treated with 5 μg/mL lipopolysaccharide (LPS) (denoted as TCP + LPS) (Sigma Aldrich; L4391). LPS was replenished at regular intervals (Days 3, 5, and 7) and cell behavior was evaluated on Days 3 and 7 using ELISA and immunostaining as explained in sections 2.19, 2.20 below. A separate group was seeded into iHPN and Col hydrogels, and LPS was added to the culture medium similarly (i.e., iHPN + LPS and Col + LPS) (5 μg/mL on Days 3, 5, and 7) to evaluate the effect of biomaterials on astrocyte reactivity under inflammatory conditions.

### 2.16 In vitro RAW 264.7 cell polarization assessment

RAW 264.7 cells were generously donated by Dr. Keselowsky's lab (University of Florida). RAW 264.7 cells are monocyte/macrophage-like cells derived from the Abelson leukemia virus-transformed cell line of BALB/c mice. As mentioned previously, these cells function similarly to human macrophages, making them an appropriate model for studying macrophage activity [56]. RAW 264.7 (P 1–3) cells were cultured in a complete medium having DMEM (high glucose), 10 % FBS, and 1 % Pen-Strep at 37 °C and 5 % CO_2_. The media was changed every 2–3 days; the cells were trypsinized when the cell's confluency reached around 70–80 %. iHPN solution was neutralized as explained above and ∼1 × 10^6^ cells/group were seeded within the hydrogels (n = 3 from different donors). 150 μL of the solution was then cast into an 8 mm silicone mold and incubated at 37 °C, 5 % CO_2_ for 20 min. The hydrogels were then removed from the silicone molds and cultured in 24-well plates. The media was changed every 2 days up to 7 days. Following that, the cells were fixed and processed for CD206 and CD86 immunostaining, as explained in section 2.19. CD206 is a C-type lectin expressed predominantly by most tissue macrophages, dendritic cells, and specific endothelial cells. CD206 functions in endocytosis and phagocytosis and plays an important role in immune homeostasis by scavenging unwanted mannoglycoprotein [51]. CD86 is a surface molecule expressed on macrophages that provides costimulatory signals necessary for T cell activation and survival [52]. Thereby, the expression levels of CD206 and CD86 markers denote macrophage polarization. M2 (anti-inflammatory) macrophages have an increased CD206 expression but decreased CD86 expression [53]. In this study, rat tail collagen type I solution (Corning; 354249) was used as a control. It was neutralized to a concentration of approximately 10 mg/mL using 1 N NaOH and 10x PBS. The same initial cell density used in iHPN was added to the neutralized collagen solution, and genipin was incorporated into the collagen solution (Col) at a final concentration of 5 mM. Cells cultured in 24-well TCP plates, at the same initial seeding density, served as controls. To induce macrophage activation, a positive control group of cells was seeded in 24-well TCP plates at the same initial seeding density and treated with 10 μg/mL LPS (i.e., TCP + LPS). LPS was replenished at regular intervals (Days 3, 5, and 7) and cell behavior was evaluated on Days 3 and 7 for cytokine secretion (ELISA), NO levels, and immunostaining, as explained in section 2.20 below. A separate group was seeded into iHPN and Col hydrogels, and LPS was added to the culture medium in the same way (i.e., iHPN + LPS, and Col + LPS) (10 μg/mL on Days 3, 5, and 7) to evaluate the effect of biomaterials on macrophages reactivity under inflammatory conditions.

### 2.17 In vitro pre-activated RAW 264.7 cell polarization assessment

RAW 264.7 cells were pre-activated by incubating them with 10 μg/mL of LPS for 60 min at 37 °C at 5 % CO_2_ [54]. Approximately 1 × 10^5^ pre-activated RAW 264.7 cells were added to the pre-gel iHPN solution and hydrogels were cast into 8 mm silicone molds and incubated at 37 °C with 5 % CO_2_. The hydrogels formed were then transferred into 48-well plates and 1 mL of complete media was added. The cell-conditioned media was collected on Days 3 and 7 for ELISA as explained in section 2.20 below. For positive control, a similar density of pre-activated cells was seeded in 48-well TCP plates. Additionally, a similar density of pre-activated cells was seeded in Col hydrogels as a control.

### 2.18 In vitro human microglial cell reactivity assessment

Human microglial cell line clone 3 (HMC3, CRL-3304) was purchased from the American Type Culture Collection (ATCC). HMC3 is widely recognized in the literature as an appropriate model for studying neuroinflammation in the CNS [55]. The cells were cultured at 37 °C with 5 % CO₂ in Eagle's Essential Medium supplemented with 1 % Pen-Strep and 10 % FBS, as recommended by the manufacturer. At 70–80 % confluency, the cells were trypsinized, and approximately 1 × 10⁵ cells per hydrogel were encapsulated within iHPN using genipin as a crosslinker, as previously described. LPS (10 μg/mL) was added to the culture media on Days 3, 5, and 7. On Days 3 and 7, the cell-conditioned media were collected for ELISA analyses. Subsequently, the HMC3 cells encapsulated in iHPN hydrogels were fixed with 4 % PFA, and immunostaining was performed to detect CD68 expression as explained in section 2.19 below. LPS stimulation is well-documented to activate HMC3 cells, leading to increased pro-inflammatory cytokine production and upregulation of CD68 expression [56].

### 2.19 Immunostaining cells in iHPN

After the specific incubation times (Days 3 and 7), the cells in the hydrogels were fixed using 4 % PFA for 1 h at RT. The hydrogels were then permeabilized using 0.2 % triton X-100 for 15 min, followed by blocking using 5 % bovine serum albumin for 1 h at RT. Primary antibodies were added and incubated for 15 h: β III tubulin (1:1000, Invitrogen; PA5-85609), CD206 (1:1000, Abcam; ab64693), CD86 (1:1000, Abcam; ab238468), CD68 (1:500, Abcam; ab955), and GFAP (1:1000, Invitrogen; OPA1-06100). The hydrogels were then washed using phosphate-buffered saline with Tween 20 (0.2 % v/v) (PBST) three times, 5 min each. Secondary antibody Goat anti-Rabbit IgG (H + L) Highly Cross-Adsorbed Secondary Antibody, Alexa Fluor™ 488 (Invitrogen; A-11034) or Goat anti-Mouse IgG (H + L) Highly Cross-Adsorbed Secondary Antibody, Alexa Fluor™ 647 (Invitrogen; A-21236) at 2 μg/mL was added and incubated at RT for 1 h. The hydrogels were then washed using PBST three times, 5 min each. DAPI (1 μg/mL) was used as a counterstain. The hydrogels were subsequently washed using PBST once for 5 min. The cells were then imaged using a Zeiss 880 confocal microscope. Z stack images were acquired and processed using Zeiss (black edition) software. The image intensity was quantified using Zeiss Blue software. As noted above, CD206 expression is related to the M2 phenotype (anti-inflammatory) of macrophages, whereas CD86 expression correlates to the M1 phenotype (inflammatory) of macrophages [57].

### 2.20 ELISA quantification

The conditioned media obtained at different time points were collected and frozen at −80 °C until further use. IL-4, TNF-α and IL-6 levels were estimated individually from RAW 264.7 cells conditioned media using Biolegend's LEGEND MAX™ ELISA kit (IL-4: 431107, TNF-α: 430907, IL-10: 431417) and human IL-1β was estimated using human astrocyte cell conditioned media and Biolegend's LEGEND MAX™ ELISA kit (IL-1β: 437007). Similarly, at Days 3 and 7 of HMC3 culture within iHPN, the cell conditioned media was collected, and IL-4 (LEGEND MAX™ Human IL-4 ELISA kit, 430307); TNF-α (LEGEND MAX™ Human TNF-α ELISA kit, 430207) and IL-1β (LEGEND MAX™ Human TNF-α ELISA kit, 437007) levels were estimated. On the day of the experiment, the cell culture media supernatant was thawed at RT and centrifuged at 400×*g* for 10 min at 4 °C, to pellet down any debris. The collected supernatant was further used for ELISA as per the manufacturer's instructions. All data points were run in duplicate, and the two readings for each sample were averaged. The averages and standard deviations were calculated for all replicates. The cytokine levels were determined in terms of pg/mL concentration based on the standard curve generated using the kit standards.

### 2.21 Griess assay

Nitric oxide (NO) production was determined by measuring its stable product nitrite, which is formed by the spontaneous oxidation of NO under physiological conditions [58]. The nitrite levels in sample supernatants of the RAW 264.7 cells were quantified at different time points using a Griess reagent kit (Thermo Scientific, G7921). Briefly, 150 μL of conditioned media was added to 96-well plates. To that, 20 μL of Griess reagent and 130 μL of deionized water were added. The solutions were then incubated for 30 min at RT. The absorbance of the sample was measured at 548 nm. The nitrite concentration was then quantified from the standard provided in the kit.

## 3 Statistical analysis

All experiments were performed with n = 3 in each group, and data were analyzed using Graph Pad Prism. Wherever applicable, the One-Way ANOVA Tukey's *t*-test was applied, or an unpaired *t*-test was used to analyze significant differences between the groups. p < 0.05 was considered a significant difference between groups.

## 4 Results and discussion

### 4.1 Efficacy of modified Hudson protocol to decellularize human nerves

**Fig. 2:**
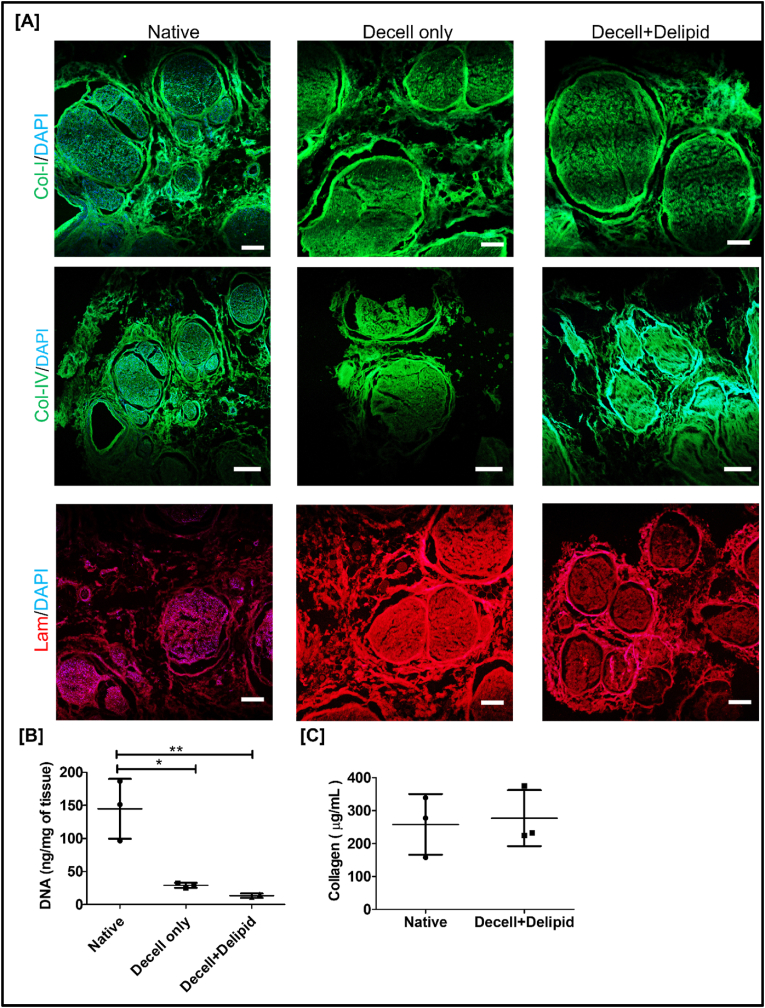
**Efficacy of modified Hudson protocol for decellularization of human nerves:** A) Representative immunostaining images for Col-I (green), Col-IV (green), and Lam (red) in native, decell only, decell + delipid nerves (Scale bar: 200 μm for Col-I and Lam; 500 μm for Col-IV images). The nerves were counterstained with DAPI (blue); B) Residual DNA quantification in native, decell only, decell + delipid nerves (n = 3, data were analyzed using One Way ANOVA, Tukey's *t*-test, ∗∗p < 0.05); C) Collagen estimation in native fresh and decell + delipid nerves (n = 3, data were analyzed using unpaired *t*-test). (For interpretation of the references to color in this figure legend, the reader is referred to the Web version of this article.)

The efficacy of the modified Hudson protocol for cellular removal and ECM retention was assessed using DAPI stain to quantify nuclear debris and antibody staining for collagen I (Col-I), collagen IV (Col-IV), and laminin (Lam) to assess ECM retention. As shown in Fig. 2A, the decell only and decell + delipid human sciatic nerves retained important ECM components, Col-I, Col-IV, and Lam, comparable to native fresh nerves. The presence of ECM proteins like Col-I, Col-10.13039/501100000026IV, and Lam in decellularized nerves has been shown to support cellular adhesion, proliferation, and neuronal regeneration [59]. Additionally, the absence of DAPI staining in decell only and decell + delipid nerves indicates the removal of cellular nuclei (Fig. 2A). We also confirmed the residual DNA content. As shown in Fig. 2B, the DNA content in decell-only tissue was approximately 23 ng/mg, while in decell + delipid nerves, the DNA content was further reduced to about 8 ng/mg. Washing decell nerves with ethanol resulted in further cellular/DNA removal, as the latter is known to lyse cells by dehydrating the tissue [60]. Furthermore, the residual DNA content in decell only and decell + delipid nerves are both compatible with the current decellularization guidelines of <50 ng dsDNA/mg of tissue, proposed by Crapo et al. [61]. We further estimated collagen content in decell + delipid and native fresh nerves. As shown in Fig. 2C, we did not find any significant differences in collagen content between native fresh and decell + delipid nerves. On estimating the endotoxin levels, we found our decell + delipid nerves had endotoxin levels of 0.3 ± 0.23 EU/mL, which is well below the acceptable limit reported in previous literature [62]. Overall, these results indicate that the modified Hudson method effectively removes cellular content while retaining the ECM during the decellularization of human sciatic nerves. These decellularized nerves were then used to fabricate injectable hydrogels following delipidation, as explained below.

### 4.2 Efficacy of dichloromethane: ethanol solvent on delipidation of decellularized human nerves and its effect on pepsin digestion

**Fig. 3:**
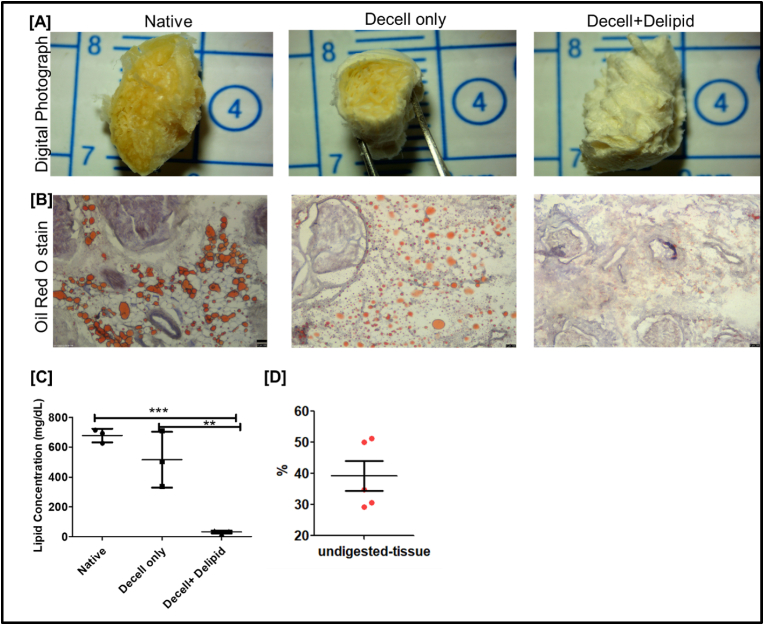
**Lipids and percentage of decellularized nerve digestion quantification:** A) Representative digital images of lyophilized nerves; B) Oil Red O staining (red droplets) for native, decell only, decell + delipid nerves (Scale bar = 200 μm); C) Lipid estimation in native fresh, decell only and decell + delipid nerves (n = 3, data were analyzed using One Way ANOVA, Tukey's *t*-test)**;** D) Percentage of undigested tissue following the pepsin digestion. (For interpretation of the references to color in this figure legend, the reader is referred to the Web version of this article.)

The presence of lipids in decellularized tissue has been shown to hinder hydrogel gelation [63,64]. Previously, using decellularized and delipidated porcine peripheral nerves, we demonstrated that lipids in decellularized peripheral nerve tissue impair the gelation kinetics and mechanical stiffness of the hydrogel [32]. Therefore, here, we studied the efficiency of delipidation for decellularized human peripheral nerves using either (i) hexane: isopropanol (3:1 v/v) or (ii) dichloromethane: ethanol (2:1 v/v) solvent. We found that both solvent mixtures efficiently reduce lipid content in decellularized human peripheral nerves (Supplementary Fig. S1). As shown from Oil Red O staining and lipid quantification, nerves delipidated with hexane: isopropanol (3:1 v/v) contained higher lipid content compared to those treated with dichloromethane: ethanol (2:1 v/v) and did not undergo subsequent pepsin digestion (Supplementary Fig. S2). Therefore, for further studies, we used only nerves delipidated with dichloromethane: ethanol (2:1 v/v). This solvent has been shown to effectively reduce lipid remnants in various decellularized tissues [32,64,65]. As shown in Fig. 3, nerves subjected to dichloromethane and ethanol after decellularization had significantly lower lipid content compared to native fresh or decell-only nerves. Digital photographs of the nerves show that the presence of lipids (yellowish areas, Fig. 3A) decreases after decellularization and delipidation. This was further confirmed by Oil Red O staining and lipid quantification using sulfo-vanillin reagent (Fig. 3B and C). Oil Red O staining shows that decellularized nerves had reduced lipid content compared to native fresh nerves. Similar results were observed when estimating total lipids using sulfo-vanillin reagent, where decell + delipid nerves showed significantly lower lipid content compared to native and decell only nerves (Fig. 3B and C). We have observed similar results for decellularized porcine peripheral nerves [32]. We further investigated the efficiency of pepsin digestion for decell + delipid human nerves. Initially, we performed pepsin digestion using three different decell + delipid tissue concentrations: 10, 15, and 20 mg/mL. We observed that a concentration of 10 mg/mL was too fluid and unable to form a stable hydrogel, whereas 20 mg/mL was too viscous to handle. The 15 mg/mL concentration was selected because it had optimal viscosity and could form stable hydrogels. Furthermore, at 15 mg/mL, we found that approximately 40 % of the decell + delipid nerve was not digested by pepsin, indicating that around 60 % of the decell + delipid nerve was completely digested (Fig. 3D). These results are aligned with previous literature, which reports that decellularized human tissues are not fully digested by the pepsin enzyme [66,67].

### 4.3 Rheological and biodegradation analysis of iHPN

**Fig. 4:**
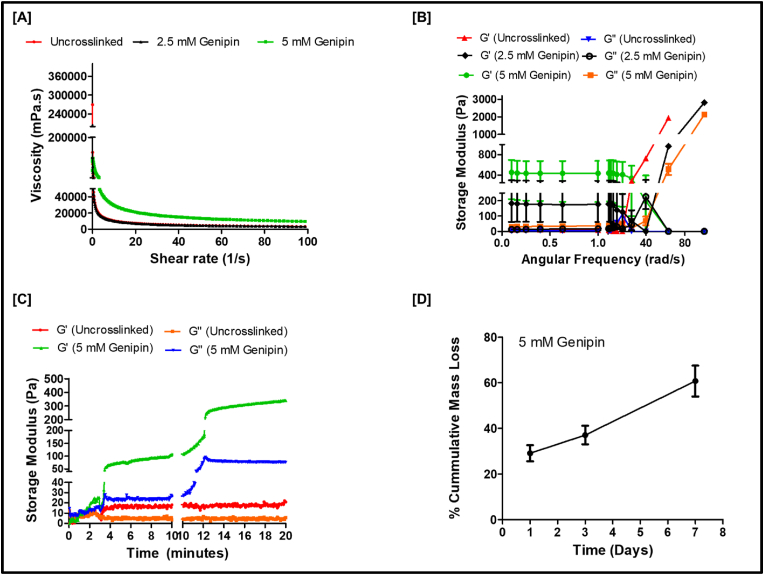
**Rheological and *in vitro* biodegradation assessment of iHPN:** A) Shear-thinning assessment of iHPN (uncrosslinked, 2.5 and 5 mM genipin-crosslinked) demonstrating injectability; B) Frequency sweep of iHPN (uncrosslinked, 2.5 mM and 5 mM genipin-crosslinked) from 0.1 to 100 rad/s angular frequency and 1 % strain; C) Time sweep of iHPN (uncrosslinked and 5 mM genipin-crosslinked) at 1 % strain, 1 Hz at 37 °C; D) *In vitro* biodegradation of iHPN (5 mM genipin-crosslinked) using 1x PBS at 37 °C.

Genipin crosslinking was used to further adjust the mechanical properties of the hydrogels, aiming to match the final mechanical stiffness to that of native CNS tissue. We initially tested two different concentrations of genipin, 2.5 and 5 mM, for iHPN crosslinking. The injectability of hydrogels was evaluated by assessing their shear-thinning properties using a rheometer [68]. The viscosity of both uncrosslinked iHPN and genipin-crosslinked iHPN decreased with an increase in shear stress (Fig. 4A), indicating the injectability of the iHPN solution. We further confirmed the injectability by passing the pre-gel solution through needles of different gauge sizes (25 G, 27 G, and 30 G). As shown in **Supplementary Videos 1**–**3** (SV1-SV3), both 25 G and 27 G needles were able to successfully aspirate the pre-gel solution from the microcentrifuge tube and allowed for smooth passage. However, while the 30 G needle was unable to aspirate the solution, the solution was still able to pass through the needle when external pressure was applied (Supplementary Table ST1). Therefore, the iHPN solution can be administered using 25 G and 27 G needles, which are widely used for biomacromolecule delivery in CNS treatments [69].

The mechanical stiffness of the hydrogel was assessed using a rheological frequency sweep study [70], which examined the effect of frequency on the viscoelastic properties of the hydrogels at a fixed shear strain of 1 %. At higher angular frequencies, the G′ of genipin-crosslinked iHPN remained stable up to 60 rad/s, but an increase in G′ was observed after 40 rad/s (Fig. 4B). We found that iHPN crosslinked with 2.5 mM of genipin exhibited a mechanical stiffness (G′) of around 200 Pa at 1 rad/s angular frequency; however, the hydrogels were too soft and liquid-like to handle (Fig. 4B). The G′ of uncrosslinked iHPN was around 10 Pa at 1 rad/s, while 5 mM genipin-crosslinked iHPN exhibited a G’ of approximately 400 Pa at 1 % rad/s, indicating that genipin-crosslinked iHPN had significantly higher mechanical stiffness compared to uncrosslinked gels (Fig. 4B). Hydrogels with a storage modulus of 400 Pa have been shown to promote increased neurite outgrowth [71]. Therefore, as 5 mM genipin-crosslinked iHPN had sufficient mechanical stiffness for neural tissue regeneration, only 5 mM genipin-crosslinked hydrogels were used for further studies.

The gelation kinetics of the hydrogels were studied using rheometer time sweep analysis. As shown in Fig. 4C, both uncrosslinked iHPN and iHPN crosslinked with 5 mM genipin began to gel within 3 min of incubation at 37 °C, as indicated by the increase in G′ over G’’ [72]. After 12 min of incubation at 37 °C for both uncrosslinked iHPN and genipin-crosslinked iHPN, G’ reached a plateau, indicating complete gelation [73].

**Fig. 5:**
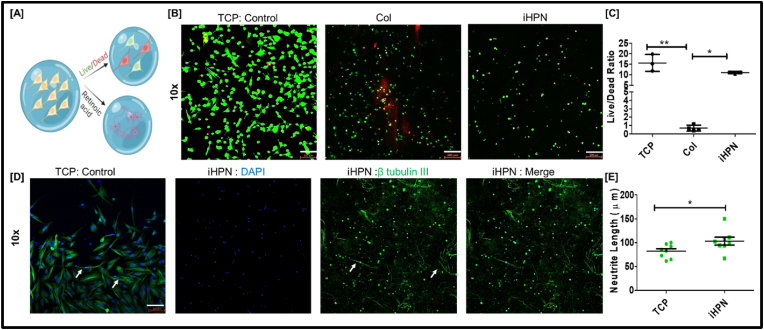
**iHPN effect on SH-SY5Y cell biocompatibility and neuronal differentiation:** A) Schematic representation for analyzing iHPN on SH-SY5Y cell viability and neuronal differentiation; B) Live/dead stain images after 3 days of SH-SY5Y cell culture on tissue culture plastic (TCP), iHPN, or Col control. Live cells are shown in green and dead cells are shown in red (Scale bar: 100 μ m); C) Live/dead ratio of SH-SY5Y cells as estimated using Image J software (n = 3). Data was analyzed using One Way ANOVA, and Tukey's *t*-test; D) Immunostaining for β III tubulin (green) to analyze neuronal differentiation of SH-SY5Y cells. DAPI (blue) was used as a counterstain. The white arrows indicate samples of neurite outgrowth in TCP and iHPN (Scale bar: 100 μm); E) Neurite length (in μm) estimated using Image J software (n = 8). (For interpretation of the references to color in this figure legend, the reader is referred to the Web version of this article.)

We further studied the biodegradation of iHPN crosslinked with 5 mM genipin. Since uncrosslinked iHPN did not have sufficient mechanical stiffness, it was excluded from the biodegradation study. As shown in Fig. 4, Fig. 5 mM genipin-crosslinked iHPN degraded approximately 60 % within 7 days when subjected to 1x PBS *in vitro*. This demonstrates that, even after genipin crosslinking, iHPN remains degradable in PBS, making it a suitable biodegradable hydrogel with mechanical stiffness appropriate for supporting neuronal cell growth.

### 4.4 Effect of iHPN on human SH-SY5Y and human brain neurons neuronal cell proliferation and differentiation

The effect of genipin-crosslinked iHPN on SH-SY5Y cell viability was evaluated using live/dead staining, as well as its influence on neuronal differentiation (Fig. 5A). As shown in Fig. 5B and C, cells cultured on genipin-crosslinked iHPN and on TCP exhibited similar viability, with no significant differences observed. This indicates that the iHPN did not exhibit cytotoxicity that would impair cell growth. However, the viability of SH-SY5Y cells in Col hydrogels crosslinked with similar genipin concentrations showed significantly higher toxicity compared to iHPN and the TCP control. This higher toxicity in Col hydrogels may be attributed to their greater mechanical stiffness compared to iHPN (Fig. 5B and C). Moreover, SH-SY5Y cells in both iHPN and Col hydrogels exhibited rounded morphologies, which can be attributed to the mechanical stiffness of the hydrogels. Previous studies have shown that SH-SY5Y cells cultured on softer matrices tend to adopt a rounded morphology, whereas cells spread out more on stiffer matrices [74]. In addition to mechanical stiffness, the rounded morphology of human SH-SY5Y cells could also be influenced by the 3D configuration of the hydrogel [75]. Since SH-SY5Y cells grown in genipin-crosslinked Col hydrogels did not show significantly higher viability, we chose not to conduct a neuronal differentiation study using Col hydrogels.

The differentiation of SH-SY5Y cells into neurons was confirmed through immunostaining for β III tubulin, a cytoskeletal protein primarily found in neurons. Undifferentiated SH-SY5Y cells typically lack β III tubulin expression, so the presence of this marker serves as an indicator of successful neuronal differentiation [76]. As shown in Fig. 5D and E, iHPN promoted enhanced neuronal differentiation of SH-SY5Y cells, with longer neurite extensions compared to cells cultured on TCP controls. Additionally, as shown in Supplementary Fig. S4, human brain neurons cultured within iHPN had significantly higher β-tubulin III expressions as compared to the Col and TCP control groups. As human brain neurons do not proliferate *in vitro*, their cellular viability was not tested and compared with TCP or Col hydrogels. Collectively, it is evident that iHPN can promote neuronal differentiation that can aid neural tissue regeneration.

### 4.5 Effect of iHPN on human astrocyte GFAP expression

**Fig. 6:**
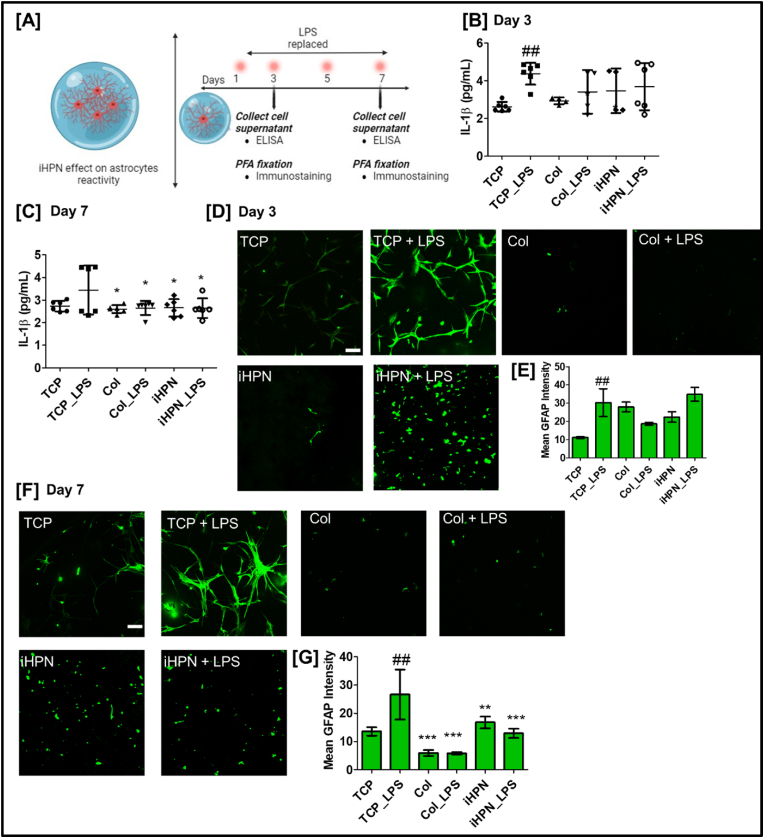
**iHPN effect on human astrocyte reactivity:** A) Schematic representation for the overall experimental plan for assessing iHPN effect on human astrocyte polarization; Bar graph depicting IL-1β estimation using ELISA at B) Day 3 and C) Day 7; D) Immunostaining for GFAP (green) to analyze astrocyte reactivity within hydrogels at Day 3 (Scale bar = 100 μm for all images); E) Bar graph representing mean GFAP intensity at Day 3, assessed using Zeiss blue software; F) Immunostaining for GFAP (green) to analyze astrocyte reactivity within hydrogels at Day 7 (Scale bar = 100 μm for all images); G) Bar graph representing mean GFAP intensity at Day 7, assessed using Zeiss blue software. Data are analyzed using One Way ANOVA and Tukey's *t*-test (∗p < 0.05); # vs TCP; ∗ vs TCP_LPS. DAPI counterstained images are provided in Supplementary Fig. S5. (For interpretation of the references to color in this figure legend, the reader is referred to the Web version of this article.)

The influence of iHPN on astrocyte reactivity was evaluated by assessing GFAP expression through immunostaining and estimating IL-1β secretion from iHPN-cultured human astrocytes. As described in the methodology, human astrocytes were cultured within the iHPN, and LPS was replenished every 2 days until Day 7 to induce an inflammatory condition. Cells cultured in Col hydrogels served as a control. On Days 3 and 7, cell culture media were collected for ELISA analysis, and the cells were fixed for immunostaining, as shown in Fig. 6A. This approach allowed us to study the effects of iHPN on astrocyte activation and its potential to modulate the inflammatory response in the context of CNS injury. On assessing IL-1β secretion from human astrocytes cultured within iHPN, we observed that on Day 3, astrocytes in the TCP + LPS group showed significantly higher IL-1β secretion compared to those in the TCP group, indicating a reactive astrocyte phenotype. However, no significant difference in IL-1β secretion was observed between astrocytes cultured in iHPN or Col hydrogels when compared to TCP + 10.13039/501100012274LPS, suggesting that both hydrogel formulations supported a reactive astrocyte phenotype at this early time point (Fig. 6B). On Day 7, however, the results were different. Astrocytes cultured in both iHPN, and Col hydrogels exhibited significantly lower IL-1β secretion compared to astrocytes in the TCP + LPS group, indicating that the hydrogel formulations had a modulatory effect on astrocyte reactivity (Fig. 6C). This reduction in IL-1β secretion suggests that iHPN and Col hydrogels may help to dampen the inflammatory response and modulate astrocyte activation, which could be beneficial for neural tissue regeneration in the context of CNS injury.

As shown in Fig. 6D, on Day 3, astrocytes cultured on TCP and treated with 5 μg of LPS exhibited a more stellate morphology, indicating a reactive state commonly associated with CNS injury [49]. In contrast, human astrocytes cultured on TCP without LPS maintained a native, star-shaped morphology, with hypertrophic cell bodies and larger cell diameters (i.e., more cell spreading), along with a more prominent stretched appearance. Interestingly, astrocytes cultured within Col hydrogels, both with and without 10.13039/501100012274LPS treatment, displayed a rounded morphology with fewer cells present, suggesting that genipin-crosslinked Col hydrogels do not support astrocyte proliferation or promote an active, spread-out morphology.

On the other hand, astrocytes encapsulated in iHPN gels exhibited a rounded morphology with less spreading compared to those cultured on TCP, both with and without LPS treatment. This difference in morphology could be attributed to the lower mechanical stiffness of the iHPN hydrogels (∼400 Pa) compared to stiffer TCP substrates (∼1 GPa) [60]. Similarly, the mechanical stiffness (G’) of Col hydrogels was around 2000 Pa at 1 Hz frequency and 1 % strain (Supplementary Fig. 3), which could influence cell proliferation and morphology. In addition to mechanical stiffness, the high collagen concentration in Col hydrogels would have affected astrocytes morphologies [61].

Upon analysis of GFAP intensity, astrocytes treated with LPS on TCP (i.e., TCP + LPS) exhibited higher GFAP expression, indicating a reactive phenotype [62]. However, when compared to TCP + LPS astrocytes, there was no significant difference in GFAP mean intensity for astrocytes cultured in iHPN, iHPN + LPS, Col, or Col + LPS, suggesting a similar reactive phenotype (Fig. 6E). On Day 7, astrocytes in Col and Col + LPS hydrogels remained low in number and maintained a rounded morphology, suggesting limited proliferation. In contrast, cells in iHPN and iHPN + LPS hydrogels were more numerous than those in Col hydrogels, although they still displayed a rounded morphology, possibly due to the softer matrix provided by iHPN (Fig. 6F).

Upon further analysis of GFAP intensity, astrocytes in Col, Col + LPS, iHPN, and iHPN + LPS showed significantly lower GFAP expression compared to those cultured on TCP + LPS, indicating a less reactive, quiescent state. This suggests that both Col and iHPN hydrogels promote a more stable astrocyte phenotype. Interestingly, no significant difference in GFAP mean intensity was observed between astrocytes cultured in iHPN and iHPN + LPS, suggesting that even in the presence of an inflammatory stimulus, astrocytes within iHPN did not adopt a reactive phenotype, which is beneficial for tissue regeneration (Fig. 6G).

### 4.6 Effect of iHPN on the production of nitric oxide (NO) and cytokines from RAW 264.7 cells

**Fig. 7:**
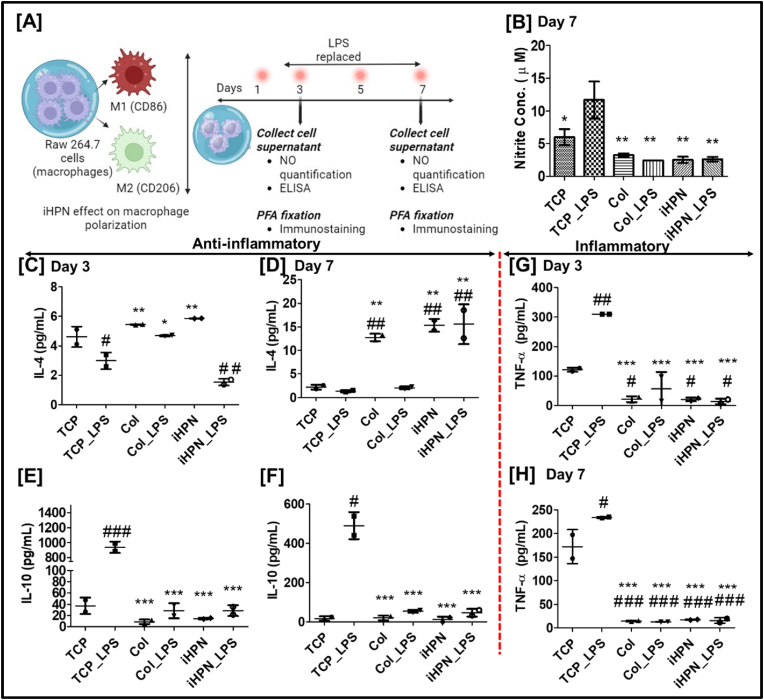
**iHPN effects on RAW 264.7 cells NO and cytokines level**: A) Schematic representation for overall experimental timeline; B) Nitrite concentration correlating NO production as estimated using Griess reagent kit; C-H) ELISA level of different cytokines (IL-4, IL-10, and TNF-α) estimated on Days 3 and 7. The samples were run duplicate. The data were analyzed using One Way ANOVA, and Tukey's *t*-test (n = 3); ∗ vs TCP_LPS; # vs TCP.

The effect of iHPN on macrophage polarization was investigated in the context of an LPS-mediated inflammatory environment. RAW 264.7 cells, a murine macrophage cell line, were cultured within genipin-crosslinked Col hydrogels, which served as a positive control. RAW 264.7 cells were cultured within both iHPN and Col hydrogels, and LPS was replenished every two days until Day 7 (Fig. 7A). Conditioned media were collected on Days 3 and 7 for ELISA analysis of cytokines (IL-4, IL-10, and TNF-α). LPS-stimulated inflammatory macrophages are known to produce higher levels of NO and pro-inflammatory cytokines, such as TNF-α [63]. NO levels were estimated using Griess reagent, by indirectly measuring the nitrite, a stable product of NO autooxidation [77]. Using the Griess reagent, we observed that nitrite levels in RAW 264.7 cells cultured in both iHPN and Col hydrogels on Day 7 were significantly lower compared to LPS-stimulated cells on TCP (Fig. 7B). However, no detectable nitrite levels were found in the conditioned media from any group on Day 3. Furthermore, the addition of LPS to RAW 264.7 cells cultured within iHPN, and Col hydrogels did not induce increased NO production. These findings suggest that both hydrogel formulations helped reduce the inflammatory response of macrophages, even in the presence of LPS, potentially promoting a more favorable microenvironment for tissue regeneration.

These findings were further supported by cytokine analysis via ELISA. As shown in Fig. 7C, IL-4 levels were higher in RAW 264.7 cultured in iHPN and Col hydrogels compared to TCP + LPS on Day 3. However, by Day 7, IL-4 levels in iHPN and iHPN + LPS were significantly higher than those in TCP and TCP + LPS groups. Interestingly, IL-4 levels in Col + LPS decreased on Day 7 in contrast to their elevated levels on Day 3 (Fig. 7D). Regarding IL-10 levels, LPS-stimulated macrophages in TCP + LPS exhibited higher levels on both Days 3 and 7 compared to all other groups (Fig. 7E and F). This can be attributed to the activation of ERK, p38, and JNK MAPK pathways, which are known to promote IL-10 production in LPS-stimulated macrophages [63]. Despite this, iHPN did not induce elevated IL-10 levels, suggesting that it may not activate these pro-inflammatory molecular pathways, even in the presence of LPS.

As expected, LPS stimulation in the TCP + LPS group resulted in increased TNF-α secretion, consistent with previous literature [78,79]. However, TNF-α levels in RAW 264.7 cells cultured within iHPN, and Col hydrogels remained low on both Days 3 and 7, even in the presence of LPS (Fig. 7G and H). Overall, these results suggest that iHPN does not induce inflammatory polarization in RAW 264.7 macrophages and instead maintains an anti-inflammatory state, even in an LPS-induced inflammatory microenvironment. These findings were further corroborated by immunostaining, as described below.

### 4.7 Effect of iHPN on RAW 264.7 cell polarization

**Fig. 8:**
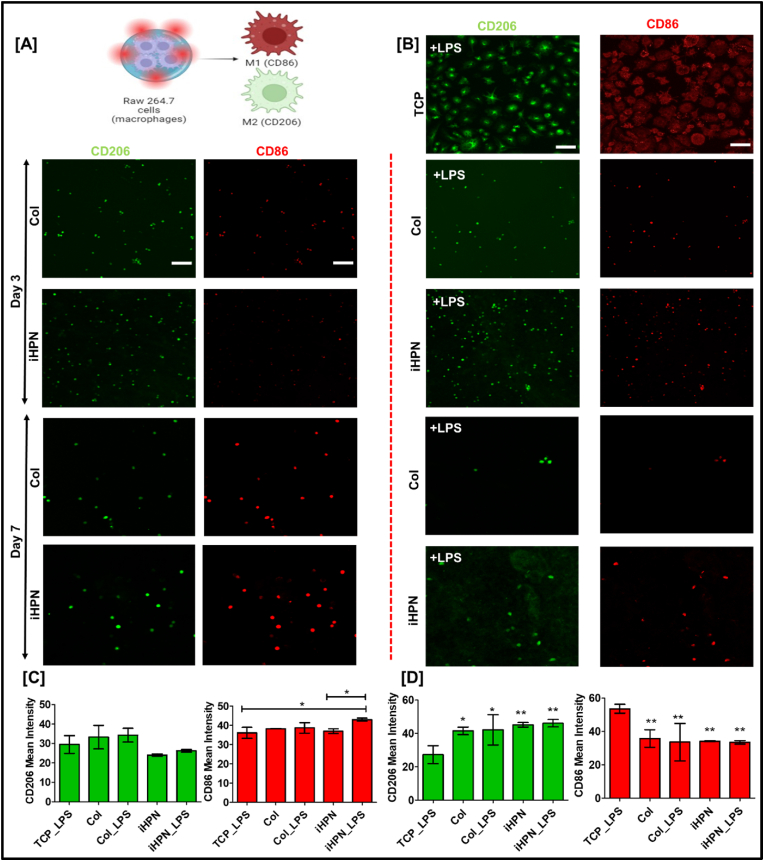
**iHPN effect on CD206 and CD86 expression from RAW 264.7 cells:** A) Schematic representation showing RAW 264.7 cells can exhibit CD86 or CD206 expression based on inherent cues; B) Immunostaining for CD206 (green) to analyze M2 polarization and CD86 (red) to analyze M1 polarization of RAW 264.7 cells within iHPN. DAPI (blue) was used as a counterstain (Scale bar = 100 μm for all images); C) CD206 and CD86 mean intensity on RAW 264.7 cells at Day 3 as analyzed using Zeiss Blue software; D) CD206 and CD86 mean intensity on RAW 264.7 cells at Day 7 as analyzed using Zeiss Blue software. Data are analyzed using One Way ANOVA, Tukey's *t*-test (n = 4), ∗p < 0.05; ∗ vs TCP_LPS. DAPI counterstained images are provided in Supplementary Fig. S6. (For interpretation of the references to color in this figure legend, the reader is referred to the Web version of this article.)

The inherent ECM cues provided by hydrogels can significantly influence macrophage polarization [80]. The effect of iHPN on macrophage polarization was further confirmed by immunostaining for CD206 and CD86 markers. Previous studies have shown that genipin-crosslinked Col hydrogels promote a higher proportion of M2 macrophages [81,82]. Therefore, in this study, RAW 264.7 cells were cultured within Col hydrogels as a positive control. As shown in Fig. 8B, LPS-stimulated RAW 264.7 cells in TCP exhibited a star-shaped morphology with pseudopod formation [[64], [65], [66]], indicative of an activated state. In contrast, RAW 264.7 cells cultured within Col and iHPN hydrogels displayed a more typical rounded morphology, suggesting they remained less activated [43]. On Day 3, there was no significant difference in the expression of CD206 and CD86 expression between cells cultured in Col and iHPN hydrogels compared to TCP + LPS, even in the presence of LPS stimulation (Fig. 8B and C). However, by Day 7, RAW 264.7 cells within iHPN showed significantly higher CD206 expression and lower CD86 expression compared to those in TCP + LPS. Furthermore, RAW 264.7 cells cultured in both iHPN, and Col hydrogels maintained higher CD206 expression, even in the presence of LPS stimulus (Fig. 8B and D). These results suggest that iHPN promotes and sustains an anti-inflammatory M2 phenotype, even under inflammatory conditions that mimic the CNS environment after injury. These findings align with our lab's previous study, which demonstrated that a similar injectable hydrogel developed using decellularized rat peripheral nerves exhibited a higher proportion of M2 macrophages in the injured spinal cord compared to Matrigel-treated animals [28].

### 4.8 Effect of iHPN on polarization of pre-activated RAW 264.7 cells

**Fig. 9:**
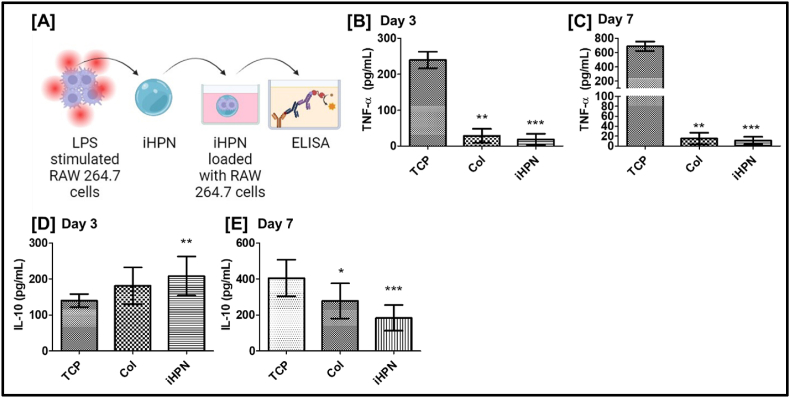
**iHPN effect on pre-activated RAW 264.7 cells:** A) RAW 264.7 cells were activated using 10 μg/mL LPS for 60 min and then incorporated into a pre-gel hydrogel solution. The hydrogels were formed by incubating the solution as explained in the methodology section earlier. ELISA was performed using the conditioned media collected on Days 3 and 7. Bar graph representing TNF-α amount secreted from TCP (n = 6), Col (n = 4), and iHPN (n = 9) cultured cells at B) Day 3 and C) Day 7. Bar graph representing IL-10 amount secreted from TCP (n = 6), Col (n = 4), and iHPN (n = 9) cultured cells at D) Day 3 and E) Day 7. ∗ vs TCP, p < 0.05.

In Fig. 7, Fig. 8, we evaluated the effect of iHPN on the polarization of RAW 264.7 macrophages externally exposed to LPS to assess iHPN's potential to maintain macrophages in a quiescent state under inflammatory conditions. Additionally, we aimed to investigate iHPN's effect on the polarization of pre-activated macrophages. Following CNS injury, it is well-documented that inflamed macrophages infiltrate biomaterial scaffolds [83]. Understanding the impact of biomaterial scaffolds on pre-activated macrophages is crucial. To investigate this, RAW 264.7 macrophages were treated with LPS to induce activation and then cultured within iHPN. As previously mentioned, LPS-activated RAW 264.7 cells secrete TNF-α and IL-10. ELISA was performed on Days 3 and 7 to measure these cytokines and assess iHPN's ability to modulate macrophage reactivity (Fig. 9A). Similar cell densities were cultured on TCP and within Col hydrogels as controls.

As shown in Fig. 9B and C, RAW 264.7 cells cultured within iHPN exhibited significantly lower TNF-α levels on Days 3 and 7 compared to LPS-activated cells cultured on TCP. No significant differences in TNF-α secretion were observed between iHPN and Col hydrogels. These findings are consistent with the data shown in Fig. 7G and H.

Interestingly, on Day 3, the IL-10 secretion from LPS-activated RAW 264.7 cells cultured within iHPN was significantly higher compared to cells cultured on TCP (Fig. 9D). However, by Day 7, IL-10 secretion in iHPN-cultured cells had significantly decreased compared to those on TCP (Fig. 9E). Collectively, these results suggest that iHPN promotes a more quiescent macrophage state in infiltrating reactive cells, which is essential for modulating the immune environment and supporting tissue repair following CNS injury.

### 4.9 Effect of iHPN on human microglial cell reactivity

**Fig. 10:**
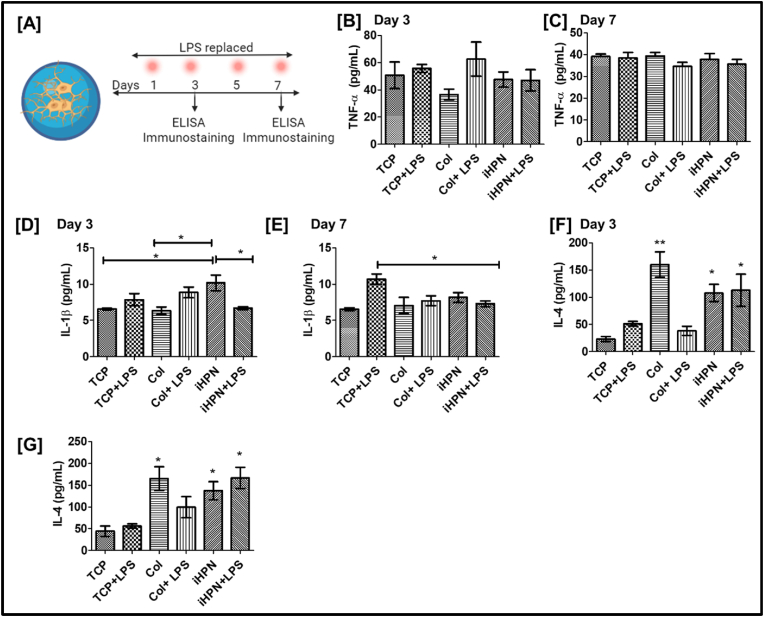
**iHPN effect on human microglial cell clone 3 (HMC3):** A) HMC3 cells were cultured within iHPN/Col hydrogels and LPS was replenished at different time points as shown in the figure above. ELISA was performed using the conditioned media collected on Days 3 and 7. Bar graph representing TNF-α amount secreted from HMC3 cultured within hydrogels estimated using ELISA on B) Day 3 and C) Day 7. Bar graph representing IL-1β amount secreted from HMC3 cultured within hydrogels estimated using ELISA on D) Day 3 and E) Day 7. Bar graph representing IL-4 amount secreted from HMC3 cultured within hydrogels estimated using ELISA on F) Day 3 and G) Day 7.

Microglial cells are the resident macrophages of the CNS and play a crucial role in maintaining CNS homeostasis by removing damaged or unnecessary neurons and synapses [84]. Microglia can exhibit opposing functions, either neurotrophic or neurotoxic, depending on their activation status. Similar to macrophages, microglial cells can polarize into M1 or M2 phenotypes. M1 microglia produces pro-inflammatory cytokines such as TNF-α, IL-6, IL-1, and IL-1β [85], whereas M2 microglia secrete anti-inflammatory cytokines and neurotrophic factors, including IL-10, IL-4, and IL-13 [86]. In this study, human microglial cell clone 3 (HMC3), a widely used cell line to study neuroinflammation, was activated using LPS. The conditioned media from these cells was collected on Days 3 and 7 for ELISA analysis, and after Day 7, HMC3 cells cultured within iHPN were fixed for immunostaining, as explained in the methodology section (Fig. 10A). As shown in Fig. 10B and C, no significant difference was observed between LPS-treated and non-LPS-treated HMC3 cells cultured on TCP, consistent with the literature suggesting that HMC3 cells do not secrete TNF-α even upon LPS stimulation. However, HMC3 cells cultured within iHPN secreted lower levels of IL-1β compared to TCP + LPS, even in the presence of LPS stimulation (Fig. 10D and E). Additionally, HMC3 cells within iHPN secreted higher levels of the anti-inflammatory cytokine IL-4 compared to TCP + LPS cultures. These results suggest that HMC3 cells maintained an anti-inflammatory phenotype even upon LPS stimulation, which was further supported by immunostaining for the CD68 marker.

**Fig. 11:**
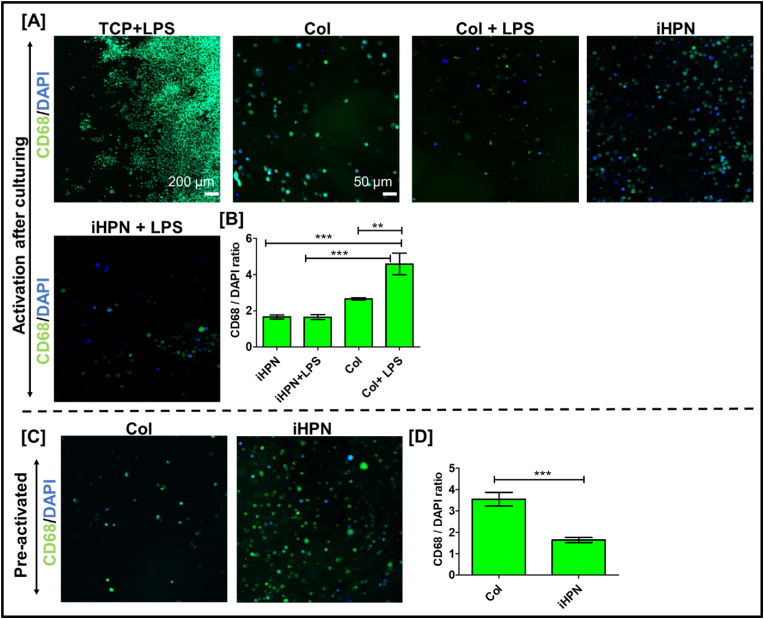
**Immunostaining assessment for analyzing the iHPN effect on HMC3:** A) Representative images for CD68 (green) and DAPI (blue) expression analysis on HMC3 cultured within hydrogels. B) Bar graph demonstrating CD68/DAPI mean intensity ratio analyzed using Zeiss Blue software. The data were analyzed using One Way ANOVA and Tukey's T-test. C) Representative images for CD68 (green) and DAPI (blue) expression analysis on LPS preactivated HMC3 cultured within hydrogels. D) Bar graph demonstrating CD68/DAPI mean intensity ratio analyzed using Zeiss Blue software. The data were analyzed using an unpaired student t-test. (For interpretation of the references to color in this figure legend, the reader is referred to the Web version of this article.)

Resting HMC3 cells do not express the CD68 marker, but its expression is upregulated after LPS activation [56,87], indicating inflammatory M1 phenotype [56,88]. Therefore, in this study, CD68 expression on HMC3 cells encapsulated within iHPN hydrogels was analyzed and compared to TCP + LPS and Col hydrogels to assess the efficacy of iHPN in maintaining the resting or anti-inflammatory phenotype of HMC3 cells in the presence of an LPS stimulus. **As shown in** Fig. 11A and B, HMC3 cells cultured within iHPN had significantly lower CD68 expression compared to TCP + LPS and Col + LPS. In addition, HMC3 cells within iHPN and Col hydrogels exhibited a rounded morphology, likely due to the three-dimensional nature of the hydrogels. HMC3 cells cultured within iHPN and stimulated with LPS (i.e., iHPN + LPS) also showed lower CD68 expression compared to Col + LPS, with no significant difference in CD68 expression between iHPN and iHPN + LPS. These results align with ELISA data mentioned earlier, which demonstrated that HMC3 cells cultured within iHPN and exposed to LPS did not secrete IL-1β, an inflammatory cytokine. Overall, our results strongly suggest that HMC3 cells cultured within iHPN maintain an anti-inflammatory phenotype even upon LPS stimulation. Additionally, HMC3 cells pre-activated with LPS and cultured within iHPN hydrogels also demonstrated similar results. As shown in Fig. 11C and D, on Day 7, HMC3 cells within iHPN displayed significantly lower CD68 expression compared to cells cultured within Col hydrogels. The precise mechanism by which iHPN sustains the anti-inflammatory phenotype of HMC3 cells requires further investigation, which is beyond the scope of this manuscript.

## 5 Discussion

Peripheral nerve grafts have been shown to support axonal regeneration and synaptic reconnection in CNS treatments in preclinical animal models, particularly for SCI [89]. However, these grafts often require complex surgical procedures, highlighting the need for a minimally invasive alternative [90]. In this study, we developed an injectable hydrogel derived from decellularized human sciatic nerves as a potential solution for CNS repair.

Various chemical methods have been explored for decellularizing human peripheral nerves [24], but the development of injectable hydrogels from decellularized human nerves remains largely unexplored. In this study, we developed an injectable hydrogel using decellularized and delipidated human sciatic nerves. The decellularization process was based on a modified Hudson method [14], where Triton X-200 was replaced with Triton X-100, and DNAase and chondroitinase ABC were added. This modification effectively removed cellular and nuclear debris while preserving key ECM components (Fig. 2) [12]. In our previous work, we observed that the presence of lipids in decellularized porcine nerves interfered with hydrogel gelation, leading to weak mechanical stiffness [32]. To address this issue, we delipidated the decellularized human sciatic nerves in this study using two different organic solvent mixtures: hexane: isopropanol (3:1 v/v) and dichloromethane: ethanol (2:1 v/v) organic solvent. Our results showed that the nerves treated with dichloromethane: ethanol (2:1 v/v) had significantly lower lipid content compared to those treated with hexane: isopropanol (3:1 v/v) treated nerves or the native/decell-only nerves (Fig. 3). Additionally, the nerves delipidated using dichloromethane: ethanol (2:1 v/v) solvent exhibited better digestion compared to those treated with hexane: isopropanol (3:1 v/v). This solvent mixture has previously been shown to effectively reduce lipids in other decellularized tissues [64,91]. The resulting digested solution was then neutralized to a pH of 7.4 to facilitate gelation. To enhance the mechanical stiffness of the hydrogels to match that of native CNS tissue, genipin was added to the neutralized solution.

Genipin is a natural crosslinker derived from the fruits of *Genipa Americana* and *Gardenia jasminoides Ellis*. Genipin can crosslink free amino groups of lysine or hydroxylysine residues of different polypeptide chains by monomeric or oligomeric crosslinks [92]. Rheological assessments demonstrated that both genipin-crosslinked and uncrosslinked iHPN solutions exhibited shear thinning properties, which are important for injectability in minimally invasive applications (Fig. 4) [44]. Additionally, the pre-gel solutions were successfully aspirated through 25 G and 27 G needles, demonstrating the potential for clinical use in various biomolecule delivery applications for CNS treatments [69].

We found that increasing genipin concentration for crosslinking raised the G′ of the hydrogel from 10 to 15 Pa (uncrosslinked) to around 400 Pa for 5 mM genipin-crosslinked hydrogels. This increase in mechanical stiffness can be attributed to the genipin crosslinking of the hydrogels. Hydrogels with G′ around 400 Pa have been shown to support optimal neurite outgrowth [71,93]. Furthermore, both uncrosslinked and genipin-crosslinked iHPN exhibited G′ values higher than G″, indicating their viscoelastic properties (Fig. 4) [[94], [95], [96]]. The viscoelastic nature of hydrogels influences cell spreading, migration, proliferation and differentiation and is therefore a crucial biological biomaterial's property to promote tissue regeneration [97].

To validate the efficacy of iHPN in supporting neural tissue regeneration, we encapsulated human SH-SY5Y cells within the hydrogel and induced neuronal differentiation using retinoic acid. The use of SH-SY5Y cells for such evaluations is well-supported by literature [98,103,104], which demonstrated the suitability of this cell line for validating hydrogel performance in neural applications. The cells cultured in iHPN gels showed similar viability compared to those cultured on TCP. As a control, we examined the cellular viability of human SH-SY5Y cells cultured in collagen hydrogels crosslinked with a similar 5 mM genipin concentration (i.e., Col). Interestingly, the human SH-SY5Y cells exhibited lower viability on Col hydrogels compared to those grown in iHPN gels, possibly due to the higher mechanical stiffness of Col hydrogels (∼2500 Pa) as comparison to iHPN hydrogels (∼400 Pa) [99].

Furthermore, upon inducing neuronal differentiation of human SH-SY5Y cells using retinoic acid, we found that the neurite lengths of human SH-SY5Y cells in iHPN were higher than those for cells cultured on TCP. We also found that the retinoic acid treatment decreased the human SH-SY5Y cell proliferation, consistent with previous literature [100]. In addition, as evident from Supplementary Fig. S4, iHPN also supported human brain neuron growth. Supplementary Fig. S4B shows that human brain neurons cultured within iHPN have significantly higher β-tubulin III expression as compared to neurons cultured within the Col hydrogel and on TCP.

The presence of pro-regenerative cues like Col-I, Lam, and growth factors within the decellularized matrix likely promote enhanced neuronal differentiation [101,102]. Laminins are particularly critical in the nervous system, playing diverse roles in both the central and peripheral nervous systems, including neuronal migration, axonal outgrowth, myelination, and neuro-muscular junction formation function [103]. Notably, laminin-coated surfaces have been reported to enhance focal adhesion kinase (FAK) protein expression in SH-SY5Y cells, which promotes improved neuronal differentiation [104]. In addition, the presence of such permissive ECM has been shown to establish a regeneration niche, supporting endogenous repair following CNS trauma*.* Our previous research findings using the Hudson method modified decellularized human peripheral nerves also shown to support neuronal outgrowth in a rat peripheral nerve injury model [105]. In addition, our lab has previously illustrated that injectable hydrogels fabricated using decellularized rat peripheral nerves support axonal regeneration in injured spinal cords of rats [29,30,106]. Mungnenast et al. have also shown the hydrogels derived using decellularized porcine spinal cord promoted human SH-SY5Y cells neuronal differentiation [76]. Similarly, Sorhun et al. have shown hydrogels derived from decellularized porcine brain supported human SH-SY5Y cells neuronal differentiation [107]. A detailed molecular mechanism for such biological effect by these decellularized scaffolds is not reported, but based on the previous literature it can be said that the iHPN shows similar effect to support neuronal cell growth and can provide a conducive matrix for neuronal differentiation.

Since CNS injury triggers a complex series of cellular events, including the activation of various immune and glial cells at the site of injury, it is important to evaluate the effect of iHPN on various immune cell phenotypes. Among the different cell types involved in neuroinflammation, astrocytes, and macrophages/microglial cells have emerged as pivotal players because of their diverse functions in the CNS [108]. Astrocytes are essential for CNS homeostasis, maintaining concentrations of key molecules (e.g., ions, metabolites, neurotransmitters), synaptic connectivity, and contributing to the formation and maintenance of the blood-brain barrier and glymphatic clearance system [109]. However, under pathological and injury conditions, astrocytes undergo morphological and molecular changes, adopting a “reactive” phenotype [48], which is characterized by cellular enlargement, increased cell division, and the upregulation of various signaling molecules [108].

Macrophages and microglial cells respond to CNS trauma, with their activation persisting for months following injury [110]. The distinct roles of macrophages in tissue repair depend on their polarization state, which is influenced by local cytokines and signaling pathways. These cells can dynamically polarize in response to the surrounding microenvironment after CNS injury, playing an important role in modulating both inflammatory and regenerative processes [111]. As such, understanding the effect of biomaterial matrices on astrocyte and macrophage/microglial polarization is essential for optimizing strategies aimed at tissue repair and regeneration.

As shown in Fig. 6, on Day 3, human astrocytes cultured within iHPN and iHPN + LPS hydrogels displayed a rounded morphology. Previous studies have shown that hydrogels with mechanical stiffness around 400 Pa result in rounded cell morphology [112]. GFAP expressions in iHPN and iHPN + LPS hydrogels were similar to the positive control (TCP + LPS), indicating a reactive astrocyte phenotype. These results also correlate with similar IL-1β secretion levels in iHPN, and Col hydrogels compared to TCP + LPS. However, by Day 7, astrocytes in iHPN exhibited GFAP expression levels like those cultured on TCP, indicating a quiescent state, which is essential for tissue regeneration (Fig. 6). Even with LPS stimulation, astrocytes in iHPN remained quiescent. This resulted in lower IL-1β secretion levels from astrocytes cultured within iHPN and Col hydrogels compared to the TCP + LPS group (Fig. 6). The presence of collagen and laminin within the decellularized matrix helps maintain the quiescent state of human astrocytes [113,114]. For example, collagen IV has been shown to downregulate GFAP expression in astrocytes by inducing thrombospondin-1 expression, a protein integral for angiogenesis and neurogenesis [115]. Furthermore, collagen IV promotes neuronal differentiation while inhibiting glial cell differentiation [116].

Human astrocytes in Col hydrogels also showed lower proliferation compared to iHPN (Fig. 6), consistent with our previous studies demonstrating that injectable hydrogels developed from decellularized rat peripheral nerves support greater astrocyte adhesion and growth than Col hydrogels [69]. Astrocytes play a crucial role in CNS regeneration by migrating to injury sites and isolating the lesion to limit the spread of inflammation [[69], [70], [71]]. However, astrocytes can become reactive in response to inflammatory cytokines at the injury site, contributing to glial scar formation, which impedes neural regeneration. Our study found that iHPN supports human astrocyte growth and maintains their quiescent state, even under inflammatory conditions. The quiescent state of astrocytes is vital for guiding axonal growth and enhancing functional recovery after spinal cord injury [117]. Overall, iHPN maintains the quiescent state of astrocytes, which is beneficial for tissue regeneration.

We further studied the effect of iHPN on macrophage cell reactivity using RAW 264.7 cell line. On Day 7, RAW 264.7 cells cultured within iHPN exhibited higher secretion of anti-inflammatory cytokine IL-4 and lower levels of pro-inflammatory cytokine TNF-α, suggesting that iHPN promotes anti-inflammatory (M2) polarization (Fig. 7). This was accompanied by lower NO secretion, even under LPS stimulation. Immunostaining revealed higher CD206 (M2 marker) and lower CD86 (M1 marker) [57] expression in iHPN and iHPN + LPS cultures compared to the TCP + LPS control (Fig. 8). Following CNS injury, reactive macrophages infiltrate biomaterials, making it crucial to understand how different biomaterials affect macrophage polarization and reactivity [118,119]. Additionally, LPS-mediated pre-activated RAW 264.7 cells cultured in iHPN secreted lower levels of TNF-α and IL-10, further supporting the conclusion that iHPN promotes an anti-inflammatory macrophage phenotype, which is critical for neural tissue regeneration [120]. Furthermore, as evident from Fig. 7, Fig. 10, RAW 264.7 and human microglial cells within iHPN hydrogels exhibited higher IL-4 secretion compared to cells grown in Col hydrogels, even in the presence of an LPS stimulus. Similarly, HMC3 cultured within iHPN exhibited lower CD68 (M1 marker) expression with lower inflammatory IL-1β and higher anti-inflammatory IL-4 cytokines secretion, even in the presence of LPS stimulation (Fig. 10, Fig. 11). Increased IL-4 secretion by macrophages and microglial cells is neuroprotective by mitigating the release of pro-inflammatory mediators such as IL-6, TNF-α, and NO [121]. The higher CD206 expression of macrophages observed within iHPN is consistent with previous literature where decellularized ECM had similar effects [122,123].

The combination of collagen fibrils, genipin, and iHPN mechanical properties likely contribute to the lower inflammatory phenotype observed in macrophages and microglial cells. Genipin has been shown to modulate macrophage phenotype towards an M2 state through the activation of the pSTAT6-PPAR gamma pathway [124]. Genipin has also been shown to reduce LPS stimulated production of TNF-α, IL-1β and NF-κB from cultured rat brain microglial cells [125].

The higher anti-inflammatory macrophage results align with our previous study, where injectable hydrogels made from decellularized rat peripheral nerves in a rodent spinal cord injury model exhibited a higher proportion of anti-inflammatory M2 macrophages compared to injury-alone animals [29]. Similarly, Young et al. demonstrated that decellularized porcine brain tissue promotes an anti-inflammatory macrophage phenotype, further supporting the potential of decellularized matrices in modulating macrophage polarization and enhancing tissue repair. While the molecular mechanisms underlying the effects of decellularized matrices on macrophage polarization remain underexplored, the literature suggests that collagen IV within these matrices promotes macrophage polarization towards the M2 phenotype via the protein kinase B (AkT) and protein kinase A (PKA) signaling pathways [126].

Moreover, decellularized nerves have been reported to contain growth factors that contribute to neuronal development. Hydrogels with optimal mechanical stiffness have been shown to promote macrophage polarization towards an anti-inflammatory phenotype [127], regulate astrocyte reactivity, and enhance neurite outgrowth. As shown in Fig. 4, genipin crosslinking in iHPN yielded a stiffness comparable to native CNS tissue, which likely contributes to these beneficial effects.

Taken together, iHPN's ECM composition and CNS-mimicking stiffness work in tandem to regulate astrocyte and macrophage reactivity while promoting SH-SY5Y and human brain neuronal differentiation. A more detailed understanding of iHPN's effects on astrocyte and macrophage/microglia reactivity, as well as neurite outgrowth, is necessary. These investigations, while beyond the scope of the current manuscript, are essential and will be pursued in future studies using iHPN in injured rat spinal cord models. In summary, the developed iHPN hydrogel matrix is biodegradable, biocompatible, and has a mechanical stiffness that matches native CNS tissue. iHPN also promotes an anti-inflammatory macrophage phenotype and maintains the quiescent state of human astrocytes, both of which are critical for CNS applications.

## 6 Conclusion

This study introduces the development of a novel in-situ gelling injectable hydrogel, injectable human peripheral nerve (iHPN), created from decellularized and delipidated human sciatic nerves. While previous research has primarily focused on decellularization techniques, the fabrication of an injectable hydrogel from decellularized nerves is a new approach. Injectable hydrogels hold significant potential as carriers for cell and drug delivery, particularly in minimally invasive procedures. In this study, the modified Hudson protocol was used for decellularization, effectively removing cellular components from the nerves while preserving key extracellular matrix (ECM) proteins like collagen I, collagen IV, and laminin. An additional delipidation step using a dichloromethane-ethanol solvent mixture significantly reduced lipid content, as confirmed by Oil Red O staining and sulfo-vanillin lipid quantification assays, enabling formation of robust hydrogels. Rheological analysis revealed that the decellularized and delipidated nerves could be digested with pepsin to form an injectable hydrogel with shear-thinning properties, which are critical for injection through small-diameter needles. When crosslinked with 5 mM genipin, a natural crosslinker, the hydrogel demonstrated mechanical stiffness suitable for supporting neuronal cells and achieved complete gelation within 10–12 min at body temperature (37 °C). The hydrogel's biodegradability was also demonstrated, with approximately 60 % degradation over seven days in PBS. Importantly, the genipin-crosslinked iHPN hydrogel supported the viability and differentiation of human SH-SY5Y cells, a neuroblastoma cell line, promoting enhanced neuronal differentiation compared to traditional tissue culture plastic (TCP). Additionally, astrocytes cultured in the hydrogel maintained a quiescent state even under inflammatory conditions stimulated by LPS, a bacterial endotoxin. This suggests the hydrogel's non-reactive properties, as evidenced by lower GFAP expression and reduced IL-1β secretion. Furthermore, macrophage polarization studies showed that RAW 264.7 cells cultured on iHPN favored an anti-inflammatory (M2) phenotype, with higher expression of CD206, reduced CD86 expression, and increased IL-4, alongside decreased TNF-α secretion. Similarly, human microglial clone 3 cells (HMC3) cultured within iHPN exhibited an anti-inflammatory phenotype, as indicated by lower IL-1β and higher IL-4 secretion, even in the presence of LPS stimulus. Additionally, HMC3 cells grown in iHPN expressed lower levels of inflammatory marker CD68, maintaining this reduced expression even in a stimulated microenvironment.

In conclusion, this injectable, in-situ gelling hydrogel derived from decellularized and delipidated human sciatic nerves shows great promise for neural tissue regeneration applications. This study has extensively demonstrated the potential of iHPN to regulate macrophage/microglial cell and astrocyte reactivity *in vitro*. Moving forward, our research will focus on leveraging iHPN as a cell delivery vehicle and assessing its impact on neuroinflammation and axonal regeneration, with an emphasis on elucidating the molecular pathways involved in rodent models of spinal cord injury.

## CRediT authorship contribution statement

**Gopal Agarwal:** Writing – original draft, Visualization, Validation, Supervision, Software, Methodology, Investigation, Formal analysis, Data curation, Conceptualization. **Kennedy Moes:** Methodology, Formal analysis. **Christine E. Schmidt:** Writing – review & editing, Supervision, Resources, Investigation, Funding acquisition.

## Data availability statement

The data that support the findings of this study are available upon reasonable request from the authors.

## Funding

The authors would like to acknowledge the funding support received by 10.13039/100000002NIH (P0225954) and Pruitt chair funding awarded to 10.13039/100006150PI Christine E. Schmidt.

## Declaration of competing interest

The authors declare the following financial interests/personal relationships which may be considered as potential competing interests: Dr. Christine E. Schmidt reports financial support was provided by 10.13039/100000002National Institutes of Health. Dr. Christine E. Schmidt has patent #PCT/US2024/024739 pending to University of Florida. the authors declares no conflict of interest If there are other authors, they declare that they have no known competing financial interests or personal relationships that could have appeared to influence the work reported in this paper.

## Data Availability

Data will be made available on request.
