# Supplementary material for: Development and in vitro evaluation of biomimetic injectable hydrogels from decellularized human nerves for central nervous system regeneration
Source: Mater Today Bio. 2025 Jan 11;31:101483. doi: 10.1016/j.mtbio.2025.101483 (PMC11787433; doi:10.1016/j.mtbio.2025.101483)
Supplement: Multimedia component 1 [file mmc1.pdf]

Development and In Vitro Evaluation of Biomimetic Injectable Hydrogels from Decellularized Human Nerves for Central Nervous System Regeneration

Gopal Agarwal[1], Kennedy Moes[1], Christine E. Schmidt[1]

[1]: J. Crayton Pruitt Family Department of Biomedical Engineering, Herbert Wertheim College of Engineering, University of Florida, Gainesville, FL, 32610, USA.

Corresponding Author:

Prof. Christine E. Schmidt,
Distinguished Professor and J. Crayton Pruitt Family Endowed Chair
J. Crayton Pruitt Family Department of Biomedical Engineering
University of Florida
Gainesville, FL 32611, United States
Email: schmidt@bme.ufl.edu

Supplementary Data

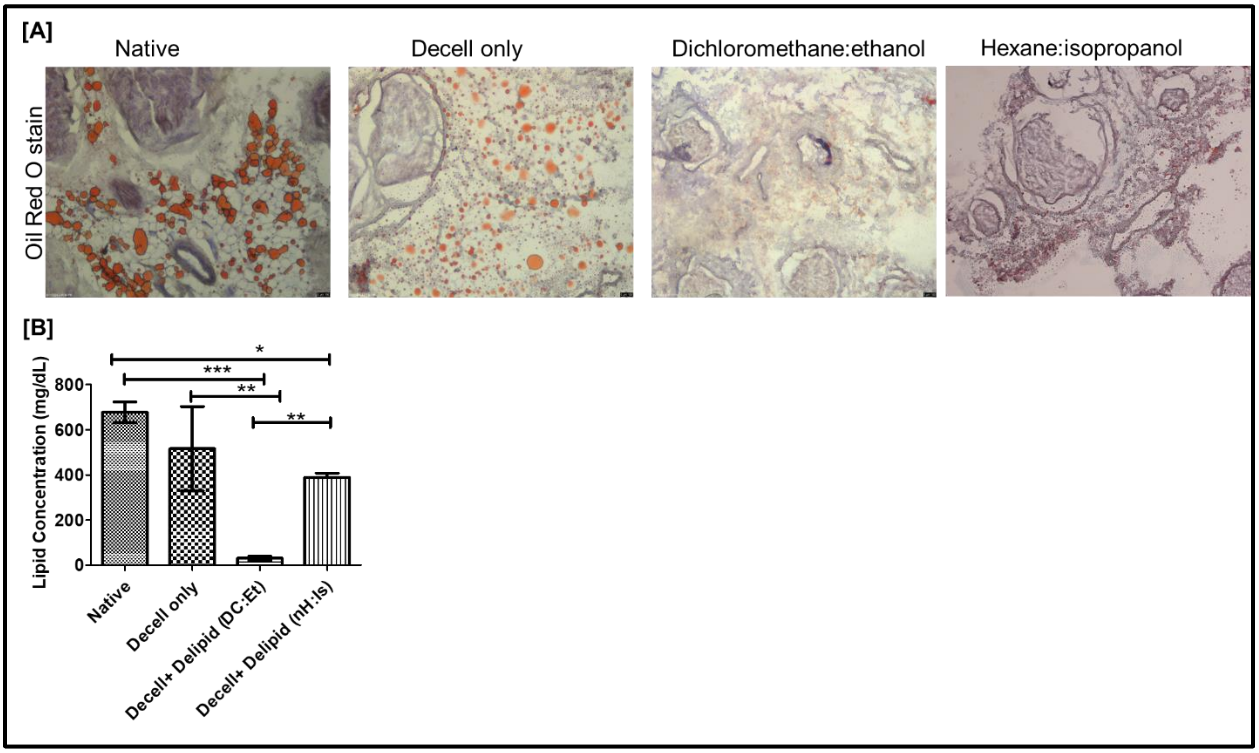

Figure S1: A) Oil Red O staining (red droplets) for native, decell only, decell+delipid nerves using dichloromethane:ethanol or n-hexane:isopropanol (Scale bar = 200 μm); B) Lipid estimation in native fresh, decell only and decell+delipid nerves using dichloromethane:ethanol or n-hexane:isopropanol (n=3, data were analyzed using One Way ANOVA, Tukey's t-test)

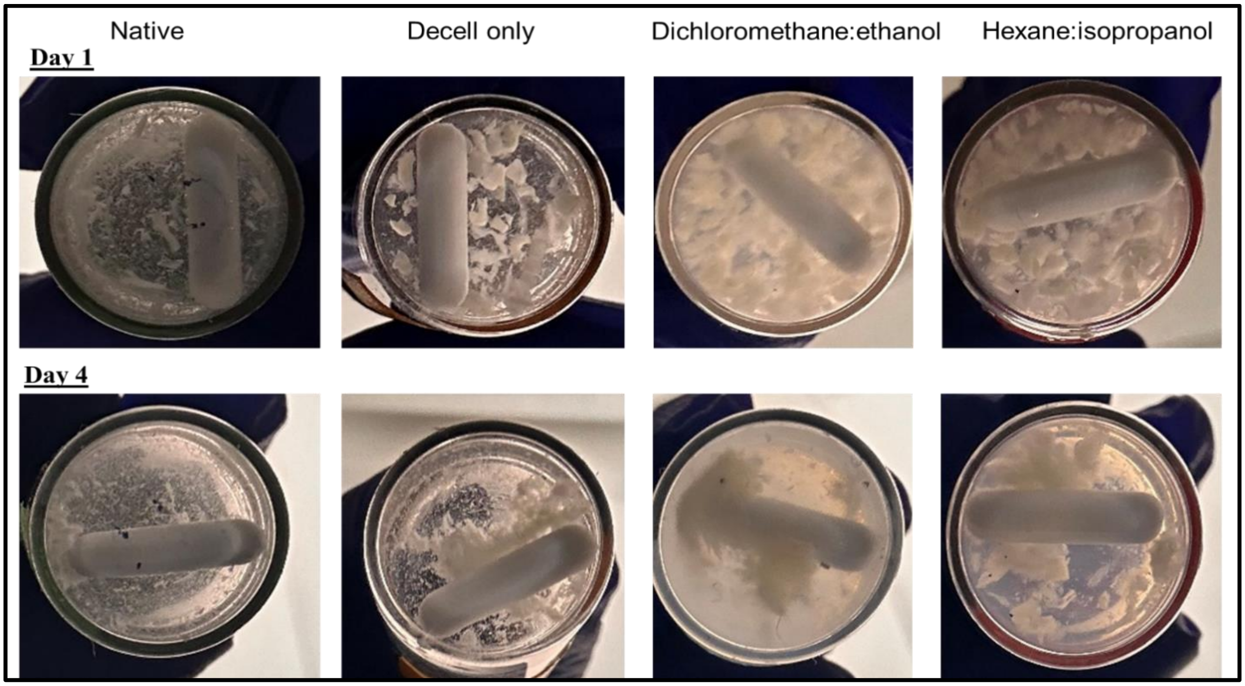

Figure S2: Representative images of nerve digestion using pepsin at different time points.

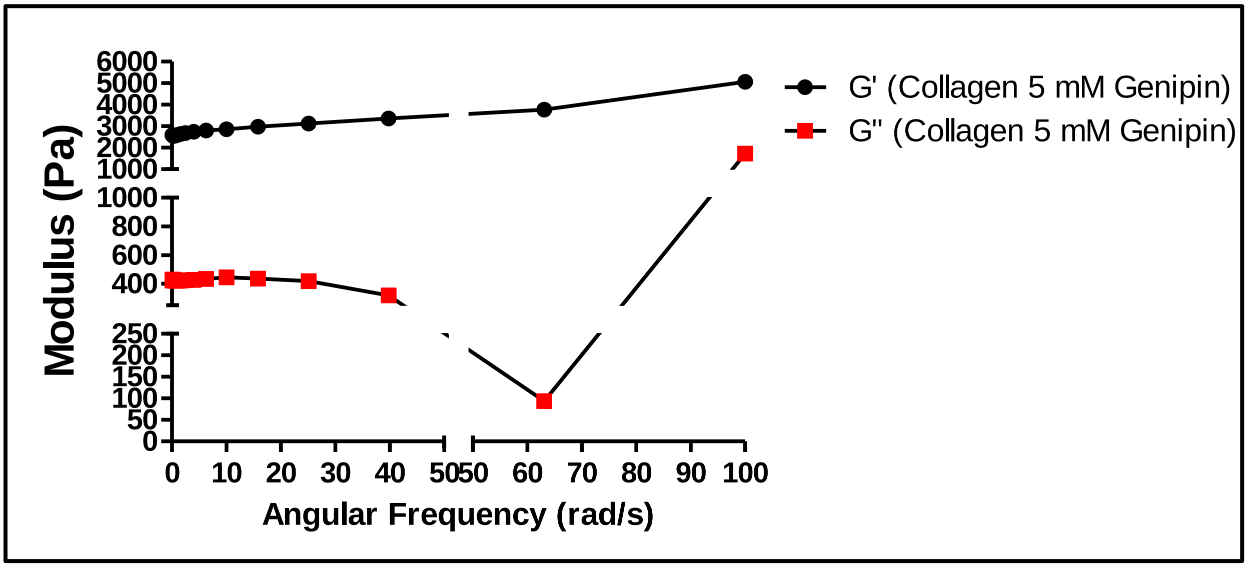

Figure S3: Representative frequency sweep of collagen (5 mM genipin-crosslinked hydrogels) depicting storage modulus (G') and loss modulus (G'').

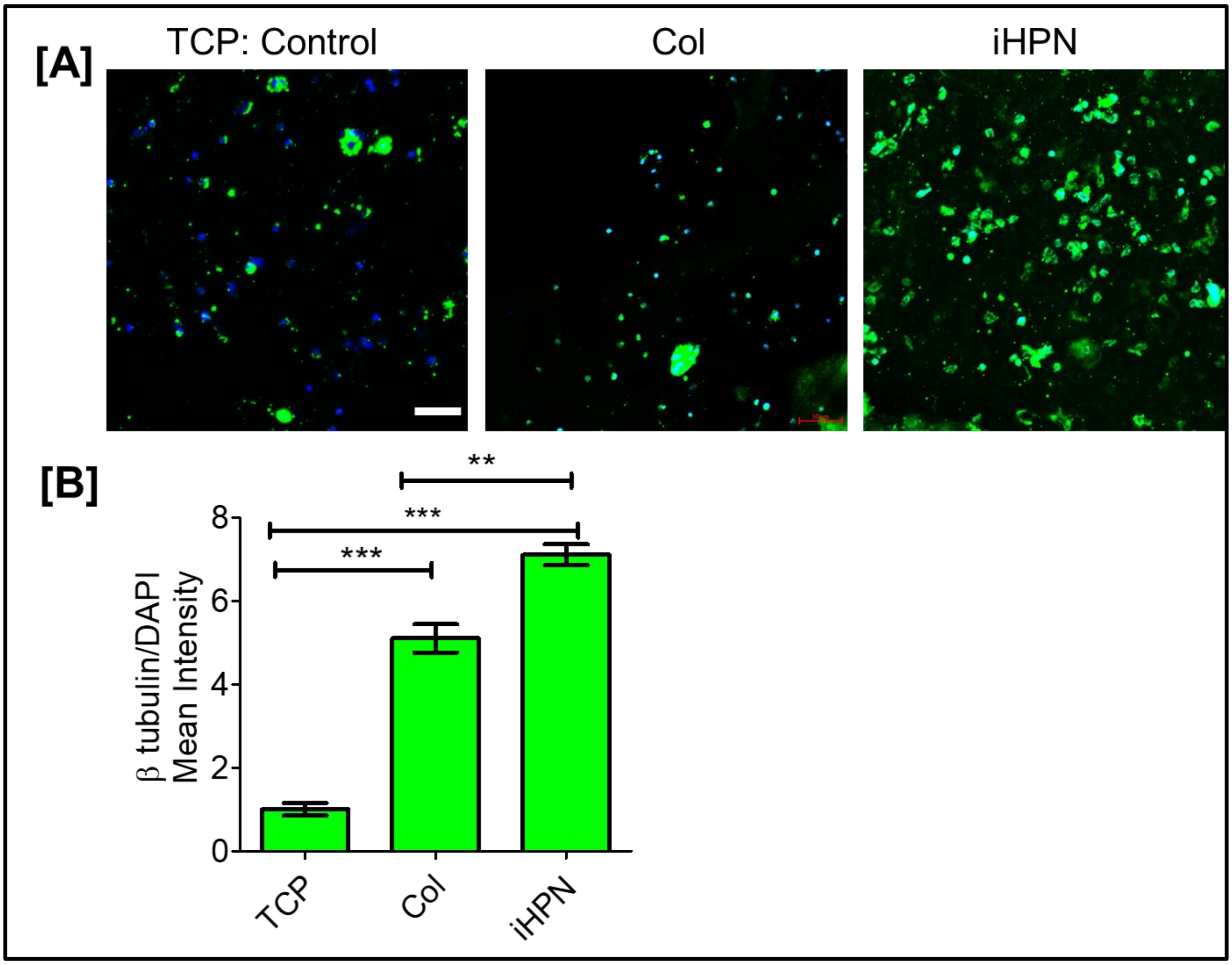

Figure S4: A) Representative immunostaining images for human brain neurons stained with β-tubulin III markers (green), counterstained with DAPI (blue) Scale bar-50 µm; B) bar graph representing mean intensity analysis for β-tubulin III. Data were analyzed using Graph pad prism, One Way ANOVA, and Tukey's t-test.

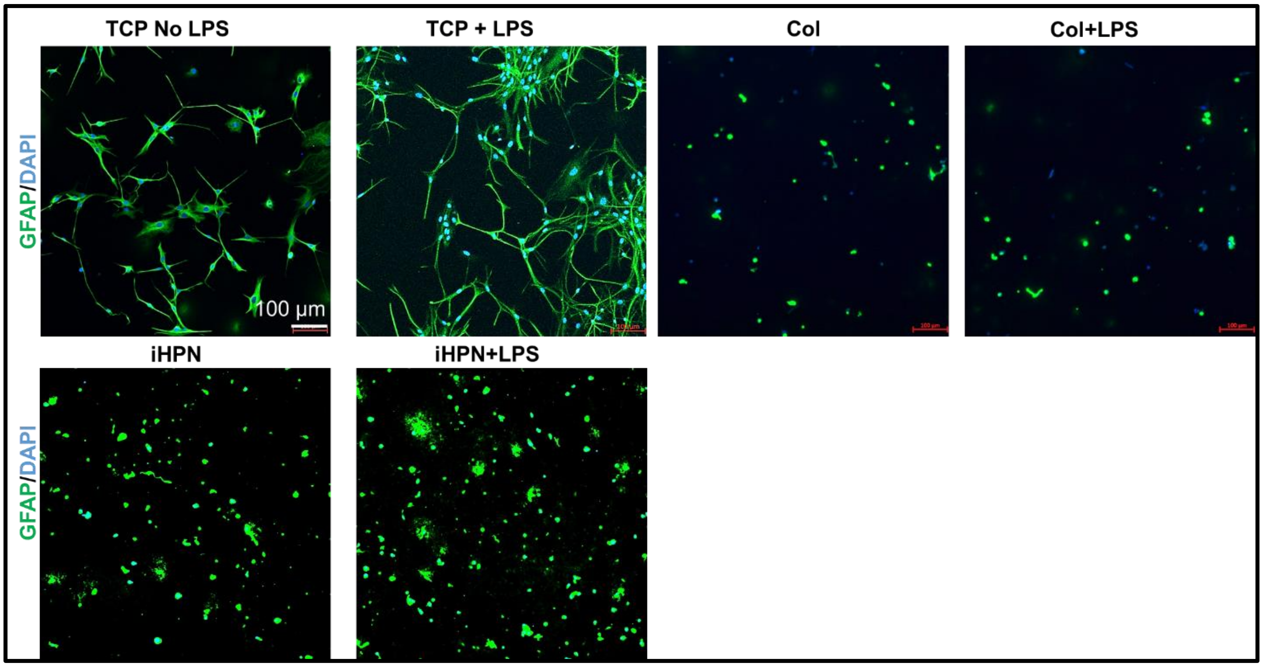

Figure S5: iHPN effect on human astrocytes reactivity: Immunostaining for GFAP (green) to analyze human astrocytes reactivity within iHPN. DAPI (blue) was used as a counterstain (Scale bar = 100 µm for all images).

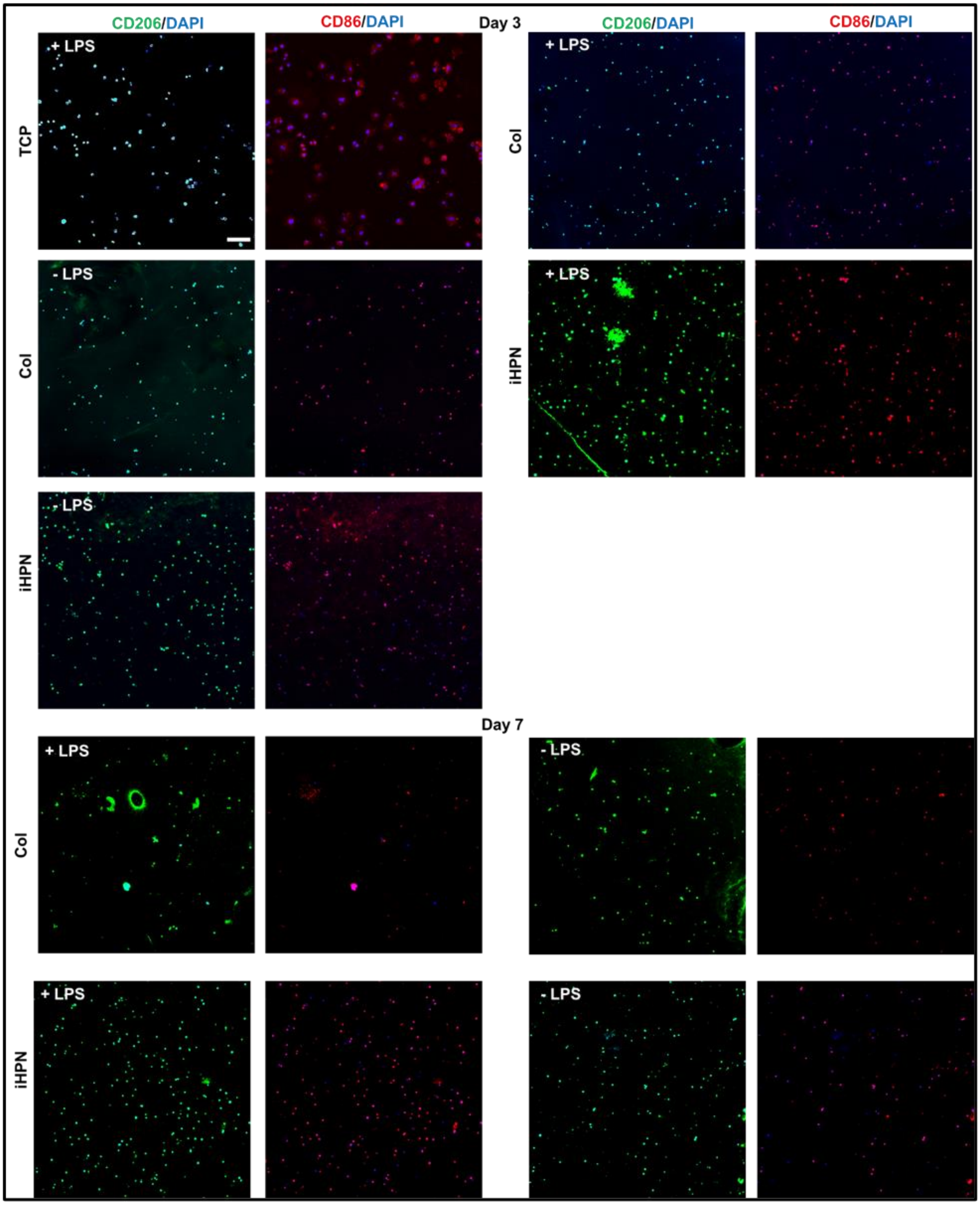

Figure S6: iHPN effect on CD206 and CD86 expression from RAW 264.7 cells: Immunostaining for CD206 (green) to analyze M2 polarization and CD86 (red) to analyze M1 polarization of RAW 264.7 cells within iHPN. DAPI (blue) was used as a counterstain (Scale bar = 100 µm for all images).
